# Genetic Variants Associated With Intraparenchymal Hemorrhage Progression After Traumatic Brain Injury

**DOI:** 10.1001/jamanetworkopen.2021.16839

**Published:** 2021-07-26

**Authors:** Ruchira M. Jha, Benjamin E. Zusman, Ava M. Puccio, David O. Okonkwo, Matthew Pease, Shashvat M. Desai, Matthew Leach, Yvette P. Conley, Patrick M. Kochanek

**Affiliations:** 1 Department of Neurology, Barrow Neurological Institute, Phoenix, Arizona; 2 Department of Neurological Surgery, Barrow Neurological Institute, Phoenix, Arizona; 3 Department of Neurobiology, Barrow Neurological Institute, Phoenix, Arizona; 4 medical student at School of Medicine, University of Pittsburgh, Pittsburgh, Pennsylvania; 5 now affiliated with Department of Medicine, Massachusetts General Hospital, Harvard Medical School, Boston; 6 Department of Neurological Surgery, School of Medicine, University of Pittsburgh, Pittsburgh, Pennsylvania; 7 Department of Critical Care Medicine, University of Pittsburgh, Pittsburgh, Pennsylvania; 8 School of Nursing, University of Pittsburgh, Pittsburgh, Pennsylvania; 9 Clinical and Translational Science Institute, School of Medicine, University of Pittsburgh, Pittsburgh, Pennsylvania; 10 Department of Pediatrics, School of Medicine, University of Pittsburgh, Pittsburgh, Pennsylvania; 11 Safar Center for Resuscitation Research, School of Medicine, University of Pittsburgh, Pittsburgh, Pennsylvania

## Abstract

**Importance:**

Intracerebral hemorrhage progression is associated with unfavorable outcome after traumatic brain injury (TBI). No effective treatments are currently available. This secondary injury process reflects an extreme form of vasogenic edema and blood-brain barrier breakdown. The sulfonylurea receptor 1–transient receptor potential melastatin 4 (SUR1-TRPM4) cation channel is a key underlying mechanism. A phase 2 trial of SUR1-TRPM4 inhibition in contusional TBI is ongoing, and a phase 3 trial is being designed. Targeted identification of patients at increased risk for hemorrhage progression may inform prognostication, trial design (including patient selection), and ultimately treatment response.

**Objective:**

To determine whether *ABCC8* (SUR1) and *TRPM4* genetic variability are associated with intraparenchymal hemorrhage (IPH) progression after severe TBI, based on the putative involvement of the SUR1-TRPM4 channel in this pathophysiology.

**Design, Setting, and Participants:**

In this genetic association study, DNA was extracted from 416 patients with severe TBI prospectively enrolled from a level I trauma academic medical center from May 9, 2002, to August 8, 2014. Forty *ABCC8* and *TRPM4* single-nucleotide variants (SNVs) were genotyped (multiplex, unbiased). Data were analyzed from January 7, 2020, to May 3, 2021.

**Main Outcomes and Measures:**

Primary analyses addressed IPH progression at 6, 24, and 120 hours in patients without acute craniectomy (n = 321). Multivariable regressions and receiver operating characteristic curves assessed SNV and haplotype associations with progression. Spatial modeling and functional predictions were determined using standard software.

**Results:**

Of the 321 patients included in the analysis (mean [SD] age, 37.0 [16.3] years; 247 [76.9%] male), IPH progression occurred in 102. Four *ABCC8* SNVs were associated with markedly increased odds of progression (rs2237982 [odds ratio (OR), 2.60-3.80; 95% CI, 1.14-5.90 to 1.80-8.02; *P* = .02 to *P* < .001], rs2283261 [OR, 3.37-4.77; 95% CI, 1.07-10.77 to 1.89-12.07; *P* = .04 to *P* = .001], rs3819521 [OR, 2.96-3.92; 95% CI, 1.13-7.75 to 1.42-10.87; *P* = .03 to *P* = .009], and rs8192695 [OR, 3.06-4.95; 95% CI, 1.02-9.12 to 1.67-14.68]; *P* = .03-.004). These are brain-specific expression quantitative trait loci (eQTL) associated with increased *ABCC8* messenger RNA levels. Regulatory annotations revealed promoter and enhancer marks and strong and/or active brain-tissue transcription, directionally consistent with increased progression. Three SNVs (rs2283261, rs2237982, and rs3819521) in this cohort have been associated with intracranial hypertension. Four *TRPM4* SNVs were associated with decreased IPH progression (rs3760666 [OR, 0.40-0.49; 95% CI, 0.19-0.86 to 0.27-0.89; *P* = .02 to *P* = .009], rs1477363 [OR, 0.40-0.43; 95% CI, 0.18-0.88 to 0.23-0.81; *P* = .02 to *P* = .006], rs10410857 [OR, 0.36-0.41; 95% CI, 0.20-0.67 to 0.20-0.85; *P* = .02 to *P* = .001], and rs909010 [OR, 0.27-0.40; 95% CI, 0.12-0.62 to 0.16-0.58; *P* = .002 to *P* < .001]). Significant SNVs in both genes cluster downstream, flanking exons encoding the receptor site and SUR1-TRPM4 binding interface. Adding genetic variation to clinical models improved receiver operating characteristic curve performance from 0.6959 to 0.8030 (*P* = .003).

**Conclusions and Relevance:**

In this genetic association study, 8 *ABCC8* and *TRPM4* SNVs were associated with IPH progression. Spatial clustering, brain-specific eQTL, and regulatory annotations suggest biological plausibility. These findings may have important implications for neurocritical care risk stratification, patient selection, and precision medicine, including an upcoming phase 3 trial design for SUR1-TRPM4 inhibition in severe TBI.

## Key Points

**Question:**

Is genetic variation in a key channel of secondary injury after traumatic brain injury (TBI) associated with patients’ risk of intraparenchymal hemorrhage (IPH) progression, a major contributor to unfavorable outcome?

**Findings:**

In this genetic association study of a prospective cohort of 321 patients with severe TBI, 8 spatially clustered *ABCC8* (sulfonylurea receptor 1) and *TRPM4* sequence variants, all brain-specific expression quantitative trait loci, were associated with IPH progression and improved clinical models; regulatory annotations further suggest biological plausibility. *ABCC8* variants that increase brain tissue *ABCC8* messenger RNA expression were associated with increased IPH progression risk, whereas variant *TRPM4* was protective.

**Meaning:**

The findings suggest that identifying patients with risk-altering genotypes of a pivotal channel in IPH progression may guide risk stratification, prognostication, patient selection, and upcoming trial design for targeted sulfonylurea receptor 1 inhibition.

## Teaser

This genetic association study examines the association of DNA sequence variability in *ABCC8* and *TRPM4* with intraparenchymal hemorrhage progression in patients with severe traumatic brain injury (TBI).

## Introduction

Hemorrhagic progression of contusions is a secondary injury process after traumatic brain injury (TBI) that is associated with unfavorable outcome and mortality.^1,2^ Hemorrhagic progression occurs in approximately 50% of patients (16%-75%), most of whom experience clinical deterioration within 24 hours.^1,2^ The risk of decompensation declines with time; few experience progression after the first week.^1,2,3,4^ The variability in reported incidence is multifactorial, relating to definitions, methodologic features, and imaging intervals.^1^ However, there are also biological differences in predispositions. Genetic differences underlying host-response and resultant secondary injury variability in TBI are increasingly recognized.^5,6,7,8^ The identification of patients who are genetically at risk for secondary intraparenchymal hemorrhage (IPH) progression has important implications for research and clinical neurocritical care.

Hemorrhage progression stems from an extreme manifestation of cerebral edema and blood-brain barrier breakdown.^2,9^ Research implicates a key role of sulfonylurea receptor 1 (SUR1)–transient receptor potential melastatin 4 (TRPM4) in this spectrum of secondary injury.^2,4,10^ SUR1-TRPM4 is a cation channel not normally expressed in the central nervous system. It is upregulated in neurovascular and glial cells after central nervous system injury.^2,4,11,12^ Channel opening results in sodium influx, oncotic edema, and cell death. Blocking SUR1-TRPM4 (via an existing drug, glibenclamide [glyburide]) has been shown to reduce cerebral edema and IPH progression and improve outcome after brain injuries such as stroke and TBI.^2^ These data led to 2 ongoing multicenter randomized trials: the phase 3 CHARM (Cirara in Large Hemispheric Infarction Analyzing Modified Rankin and Mortality) trial for stroke^13^ and phase 2 ASTRAL (Antagonizing SUR1-TRPM4 to Reduce the Progression of Intracerebral Hematoma and Edema Surrounding Lesions) trial for TBI and contusion expansion.^14^ This is in the historical context of multiple high-profile unanticipated disappointments in TBI trials testing therapies such as progesterone, hypothermia, corticosteroids, magnesium, and erythropoietin.^15,16,17,18,19^ The struggle to translate has been attributed to challenges of disease heterogeneity, treatment homogeneity, methodology, and lack of biomarkers, including genetics and imaging to guide patient selection and response.^20,21^

SUR1-TRPM4 is a unique target for both treatment and predictive and prognostic enrichment. Genetic variation may affect channel upregulation, expression, and/or function, thereby modifying risk of IPH progression. Identifying patients with high-risk genetic variants in this key (and therapeutically actionable) pathway could directly inform upcoming phase 3 trial design, patient selection, risk stratification, and prognosis. Ultimately, understanding causal variants with functional and regulatory consequences will provide insight into host and treatment response. We examined the association of DNA sequence variability in *ABCC8* (NCBI Entrez Gene 6833; encoding SUR1) and *TRPM4* (NCBI Entrez Gene 54795) on IPH progression after severe TBI. To our knowledge, this is the first study exploring the association of *ABCC8* and *TRPM4* variability with IPH progression after any central nervous system injury and the largest study of any genetic association with TBI IPH progression.^22^

## Methods

### Study Design

In this genetic association study, participants were prospectively enrolled with informed consent from health care proxies. Inclusion criteria were 16 to 80 years of age, Glasgow Coma Scale score of 8 or less on presentation (range, 3-15, with higher scores indicating less responsiveness), and more than 1 computed tomographic scan. Brain death and pregnancy were exclusionary. A total of 416 participants were enrolled consecutively from May 9, 2002, to August 8, 2014. Decompressive craniectomy affects IPH progression via a putatively distinct biology from an intact cranium^23^; we therefore a priori excluded patients with early craniectomy before follow-up imaging (n = 95) from the primary analysis, resulting in a final sample of 321 patients. The University of Pittsburgh institutional review board approved the study. This study was performed and reported in accordance with the Strengthening the Reporting of Genetic Association Studies (STREGA) reporting guideline.

### Genotyping

DNA was extracted and genotyped per established methods (eMethods in the Supplement).^5,6,7,24^ Briefly, *ABCC8* and *TRPM4* exomic single-nucleotide variants (SNVs) were genotyped using a multiplex array (Human-Core Exome, version 1.0; Illumina) with unbiased selection, including all SNVs covered by the chip (n = 25). For *ABCC8* intron coverage, 15 tag-SNVs were identified using HapMap with the tagger-algorithm pairwise approach (*r*^2^ ≥ 0.8 and minor allele frequency >0.20) and genotyped using a commercially available reagent (iPlex-Gold; Agena Bioscience) with a compact mass spectrometer (MassARRAY with Nanodispenser; Agena Bioscience). Forty SNVs were genotyped (eTable 1 in the Supplement). Genotyping was blinded to demographics and outcomes. Data cleaning and quality control included blind technical duplicates, principal components analysis, Hardy-Weinberg equilibrium testing, and exclusion of SNVs with a call rate of 95% or less.

### IPH Progression

Serial computed tomographic scans were assessed for IPH progression at presentation and at 6, 24, and 120 hours. Binary hemorrhage progression was determined using 2 criteria: thresholds using quantitative volumes of traumatic IPH (standard ABC/2 technique),^25^ and official neuroradiologist interpretations (eMethods in the Supplement). Both criteria were required to qualify as demonstrating progression to conservatively minimize false-positive associations.

### Functional Potential and Spatial Modeling

Gene expression was evaluated using the Genotype Tissue Expression (GTEx) project data portal.^26,27^ Regulatory potential was assessed using RegulomeDB, version 2.0,^28^ and HaploReg, version 4.1.^29^ Clinical significance was evaluated via systematic PubMed, Embase, and ClinVar searches. Chromosomal locations were identified using the University of California, Santa Cruz, genome browser (hg-38). The 3-dimensional SUR1-TRPM4 channel was generated using the University of California, San Francisco, Chimera program.^30^ Details are presented in eMethods in the Supplement.

### Statistical Analysis

Data were analyzed from January 7, 2020, to May 3, 2021. We compared characteristics between genotypes via analysis of variance and the Fisher exact test. Multivariable logistic models were developed with clinically relevant variables to control for confounders, including age, sex, initial Glasgow Coma Scale score, Injury Severity Score, coagulation factors, thrombocytopenia, and initial hemorrhage volume.^31,32,33,34,35^ The primary outcome was presence of IPH progression at 3 points (6, 24, and 120 hours). Patients undergoing craniectomy and quantitative IPH volume changes were included in secondary post hoc analyses. Backward-elimination models (*P* = .20 for removal; *P* = .15 for reentry) were used. Receiver operating characteristic curves were generated for IPH progression using models with clinical covariates with or without genotypes. Likelihood ratio tests compared full vs reduced models for model performance and discriminative ability. Ordinal logistic regression models for 6-month Glasgow Outcome Scale score were generated using covariates with or without genotypes (Brant tests for proportional odds across ordinal levels). Likelihood ratio tests evaluated added value of models with variants vs clinical models. Haplologit evaluated haplotype associations.^36^ Standard modes of inheritance—additive, dominance, and recessive models—were assessed. Multiple comparisons based on an unadjusted α of <0.05 were adjusted for using the established Benjamini-Yekutieli method (adjusted α = 0.00931).^37,38^ All statistical tests were 2 sided. Simulated examples evaluated the potential effect of a priori *ABCC8* and/or *TRPM4* genotype incorporation into patient selection for trial design and/or sample sizes (eMethods in the Supplement). Analyses were performed using STATA, version 15.2-16.0 (StataCorp LLC), and *P* < .05 indicated statistical significance.

## Results

Of the 321 patients included in the analysis (mean [SD] age, 37.0 [16.3] years; 247 [76.9%] male and 74 [23.1%] female; 27 [8.4%] non-White), IPH progression occurred in 102 patients, 90 within 24 hours. eTable 2 in the Supplement summarizes the clinical characteristics. The median Glasgow Coma Scale score was 7 (interquartile range, 5-7), which was representative of all patients with severe TBI at our institution during the same period (eTable 3 in the Supplement). Older age, injury mechanism, and higher presenting IPH volume were associated with increased progression (eTable 4 in the Supplement). Progression of IPH at all time points was associated with increased odds of discharge mortality by approximately 1.6- to 3.3-fold and with decreased odds of favorable functional outcome by approximately 60% to 70% (eTable 5 in the Supplement). Four *ABCC8* and 4 *TRPM4* SNVs were associated with IPH progression (eTable 6 in the Supplement).

### *ABCC8* SNVs and IPH Progression

**Figure 1.**
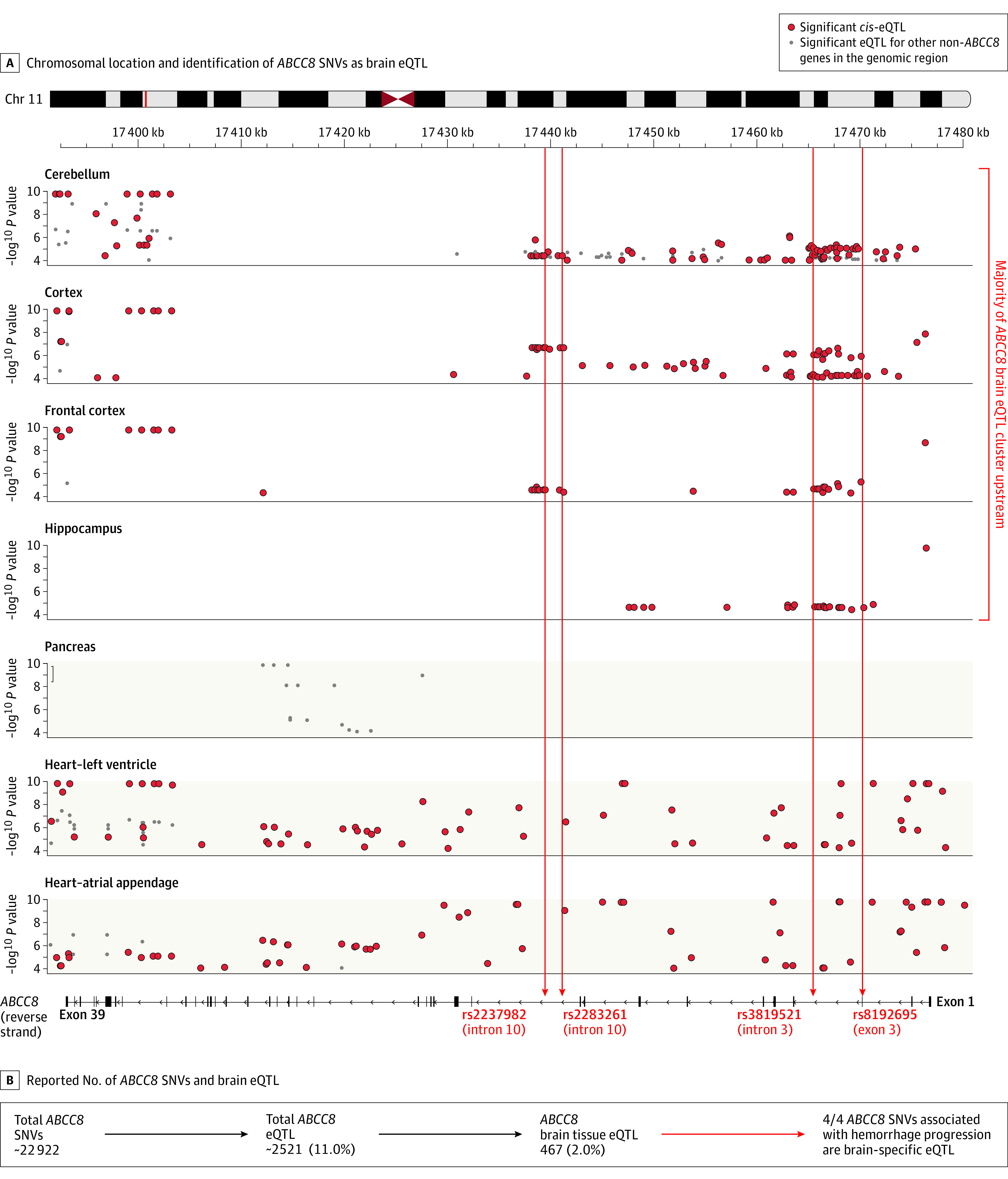
Spatial Distribution of *ABCC8* Single-Nucleotide Variants (SNVs) in Intraparenchymal Hemorrhage (IPH) Progression After Traumatic Brain Injury (TBI) A, Graph shows the −log_10_ (*P* value) of significant *cis*–expression quantitative trait loci (eQTL) within *ABCC8* (y-axis) and their location on the gene (x-axis). Subgraphs show the SNV eQTL *P* values and chromosomal locations based on different tissue isolates from the Genotype Tissue Expression (GTEx) project (brain-specific eQTL, white area; non–brain eQTL, tan area). *ABCC8* is encoded on the reverse strand; exons appear as black bars (exon 39, far left; exon 1, far right). Gray dots indicate locations and corresponding −log_10_ *P* values of *trans*-eQTL. Most brain-specific *cis*-eQTL are upstream of intron 10, including the 4 *ABCC8* SNVs associated with IPH progression (rs2237982, rs2283261, rs3819521, and rs8192695). These SNVs (red lines) are all brain-specific eQTL. No *cis*-eQTL are reported in the pancreas; all *trans*-eQTL are downstream in *ABCC8*. Both *cis*- and *trans*-eQTL are distributed evenly throughout the gene in cardiac tissue. B, Only 2.04% of *ABCC8* SNVs are brain-specific eQTL, yet all 4 *ABCC8* SNVs associated with IPH progression are brain-specific eQTL.

Three homozygous-variant intronic *ABCC8* SNVs (rs2237982, rs2283261, and rs3819521) were associated with increased IPH progression (Table and Figure 1). The fourth, rs8192695, is a synonymous coding variant. Deleterious trends emerged by 6 hours (range: odds ratio [OR], 2.73 [95% CI, 1.19-6.24] to 4.92 [95% CI, 1.42-16.97]), reached significance by 24 hours, and persisted through 120 days, most withstanding the Benjamini-Yekutieli correction (Table). Homozygous variants and heterozygote rs8192695 were associated with increased IPH progression (Table), extent of 24-hour progression (quantitative volumes, eTable 7 in the Supplement), and progression in the full cohort (eTable 8 in the Supplement). Upstream spatial clustering of these SNVs (intron/exon 3,10) is consistent with those previously reported in TBI^6,7^ and contrasts with predominantly downstream variants in glucose metabolism disorders. In this cohort, homozygous variants rs2237982, rs2283261, and rs3819521 were associated with increased intracranial pressure and cerebral edema in a blinded fashion,^5,6^ independently providing directional consistency, convergent validity, and biological plausibility.

**Table.** *ABCC8* and *TRPM4* SNVs Associated With IPH Progression in Severe TBI

SNV	Model	Genotype	Multivariable odds of IPH progression					
			6 h		24 h		120 h	
			OR (95% CI)	*P* value	OR (95% CI)	*P* value	OR (95% CI)	*P* value
***ABCC8***								
rs2237982^a^	Additive	CC	1 [Reference]	NA	1 [Reference]	NA	1 [Reference]	NA
Intron 10		CT	0.59 (0.27-1.29)	.19	0.66 (0.34-1.25)	.20	0.77 (0.41-1.44)	.41
Wild-type C		TT	1.99 (0.77-5.11)	.15	2.60 (1.14-5.90)	.02	3.23 (1.39-7.48)	.006^b^
Variant T	Recessive	CC, CT	1 [Reference]	NA	1 [Reference]	NA	1 [Reference]	NA
MAF 0.424		TT	2.73 (1.19-6.24)	.02	3.35 (1.62-6.93)	.001^b^	3.80 (1.80-8.02)	<.001^b^
rs2283261^a^	Additive	AA	1 [Reference]	NA	1 [Reference]	NA	1 [Reference]	NA
Intron 10		AC	0.73 (0.32-1.66)	.45	0.85 (0.42-1.67)	.62	0.96 (0.49-1.88)	.90
Wild-type A		CC	3.37 (1.07-10.75)	.04	3.63 (1.35-9.72)	.01	4.64 (1.66-12.97)	.003^b^
Variant C	Recessive	AA, CC	1 [Reference]	NA	1 [Reference]	NA	1 [Reference]	NA
MAF 0.404		CC	4.15 (1.47-11.66)	.007^b^	4.06 (1.68-9.72)	.002^b^	4.77 (1.89-12.07)	.001^b^
rs3819521^a^	Additive	CC	1 [Reference]	NA	1 [Reference]	NA	1 [Reference]	NA
Intron 3		CT	0.63 (0.29-1.36)	.25	0.61 (0.31-1.16)	.13	0.78 (0.41-1.48)	.45
Wild-type C		TT	2.34 (0.70-7.90)	.19	2.35 (0.84-6.52)	.10	3.43 (1.17-10.05)	.03
Variant T	Recessive	CC, CT	1 [Reference]	NA	1 [Reference]	NA	1 [Reference]	NA
MAF 0.340		TT	2.99 (0.94-9.45)	.07	2.96 (1.13-7.75)	.03	3.92 (1.42-10.87)	.009^b^
rs8192695^c^^,^^d^	Additive	GG	1 [Reference]	NA	1 [Reference]	NA	1 [Reference]	NA
Exon 3		GA	4.03 (1.14-14.2)	.03	4.31 (1.43-12.93)	.009^b^	3.06 (1.02-9.12)	.05
Wild-type G		AA	NA	NA	NA	NA	NA	NA
Variant A	Dominant	GG	1 [Reference]	NA	1 [Reference]	NA	1 [Reference]	NA
MAF 0.049		GA, AA	4.92 (1.42-16.97)	.01	4.95 (1.67-14.68)	.004^b^	3.52 (1.20-10.37)	.02
***TRPM4***								
rs3760666^c^	Additive	TT	1 [Reference]	NA	1 [Reference]	NA	1 [Reference]	NA
Intron 2		TC	0.40 (0.19-0.86)	.02	0.44 (0.24-0.83)	.01	0.47 (0.26-0.88)	.02
Wild-type T		CC	0.70 (0.14-3.42)	.65	0.48 (0.11-2.00)	.31	0.61 (0.16-2.33)	.47
Variant C	Dominant	TT	1 [Reference]	NA	1 [Reference]	NA	1 [Reference]	NA
MAF 0.335		TC, CC	0.42 (0.21-0.89)	.02	0.45 (0.24-0.82)	.009^b^	0.49 (0.27-0.89)	.02
rs1477363^c^	Additive	CC	1 [Reference]	NA	1 [Reference]	NA	1 [Reference]	NA
Intron 6		CA	0.40 (0.18-0.88)	.02	0.43 (0.22-0.81)	.01	0.41 (0.22-0.77)	.006^b^
Wild-type C		AA	0.68 (0.13-3.57)	.65	0.48 (0.11-2.15)	.34	0.61 (0.15-2.57)	.51
Variant A	Dominant	CC	1 [Reference]	NA	1 [Reference]	NA	1 [Reference]	NA
MAF 0.276		CA, AA	0.43 (0.20-0.91)	.03	0.43 (0.23-0.81)	.008^b^	0.43 (0.23-0.79)	.007^b^
rs10410857^c^^,^^d^	Additive	GG	1 [Reference]	NA	1 [Reference]	NA	1 [Reference]	NA
Intron 9		GA	0.40 (0.19-0.86)	.02	0.37 (0.20-0.70)	.002^b^	0.38 (0.20-0.71)	.002^b^
Wild-type G		AA	0.45 (0.10-2.03)	.30	0.31 (0.08-1.27)	.10	0.37 (0.10-1.37)	.14
Variant A	Dominant	GG	1 [Reference]	NA	1 [Reference]	NA	1 [Reference]	NA
MAF 0.333		GA, AA	0.41 (0.20-0.85)	.02	0.36 (0.20-0.67)	.001^b^	0.38 (0.21-0.69)	.002^b^
rs909010^c^^,^^d^	Additive	TT	1 [Reference]	NA	1 [Reference]	NA	1 [Reference]	NA
Intron 12		TC	0.27 (0.12-0.62)	.002^b^	0.29 (0.15-0.55)	<.001^b^	0.40 (0.16-0.58)	<.001^b^
Wild-type T		CC	0.81 (0.23-2.83)	.68	0.54 (0.18-1.66)	.35	0.61 (0.21-1.74)	.28
Variant C	Dominant	TT	1 [Reference]	NA	1 [Reference]	NA	1 [Reference]	NA
MAF 0.357		TC, CC	0.33 (0.16-0.70)	.004^b^	0.32 (0.17-0.60)	<.001^b^	0.34 (0.19-0.63)	.001^b^

Abbreviations: IPH, intraparenchymal hemorrhage; MAF, minor allele frequency; NA, not applicable; OR, odds ratio; SNV, single nucleotide variant; TBI, traumatic brain injury.

^a^ Previously reported to be associated with intracranial pressure and/or acute edema on computed tomography after TBI.

^b^ Statistically significant at *P* ≤ *.*00931*.*

^c^ Statistically significant (all *P* < .05; some *P* < .00931) association with volumetric hemorrhage expansion (eTable 5 in the Supplement).

^d^ Statistically significant (all *P* < .05; some *P* < .00931) association with hemorrhage progression in the entire TBI cohort, including patients undergoing craniectomy (eTable 6 in the Supplement).

### *TRPM4* SNVs and IPH Progression

Four *TRPM4* SNVs (rs3760666, rs1477363, rs10410857, and rs909010) were associated with decreased IPH progression (Table and eFigure 1 in the Supplement). Additive models revealed heterozygote advantages (range: OR, 0.27 [95% CI, 0.12-0.62] to 0.47 [95% CI, 0.26-0.88]). Protective trends emerged within 6 hours that were significant at 24 hours (range: OR, 0.29 [95% CI, 0.15-0.55] to 0.45 [95% CI, 0.24-0.82]) and 120 hours (range: OR, 0.34 [95% CI, 0.19-0.63] to 0.49 [95% CI, 0.27-0.89]). rs10410857 and rs909010 were also associated with decreased quantitative progression volumes; rs3760666 and rs1477363 had 6-hour trends (range: β, −0.27 [95% CI, −0052 to −0.02] to −0.29 [95% CI, −0.54 to −0.05) (eTable 7 in the Supplement). rs10410857 and rs909010 remained associated with decreased 24- and 120-hour IPH progression in the entire cohort, including patients undergoing craniectomy (eTable 8 in the Supplement).

### Haplotypes

*ABCC8* risk-allele haplotypes were associated with increased IPH progression at all time points (eTable 9 in the Supplement). Combining variants rs2283261 and rs8192695 yielded the strongest association, with 5.25-fold increased odds of 6-hour progression. *TRPM4* risk-allele haplotypes were associated with decreased progression at all time points; combining variants rs10410857 and rs909010 yielded the strongest association with more than 50% reduced odds of 24- and 120-hour progression (eTable 9 in the Supplement). *ABCC8* and *TRPM4* contributions appeared equivalent: given the opposing associations with progression, haplotypes combining *ABCC8* and *TRPM4* SNVs offset one another. However, haplotypes combining *ABCC8* SNVs and wild-type *TRPM4* SNVs (or vice versa) retained strong associations with IPH progression in the same direction as the individual SNVs. A minority of haplotypes (16.6%) combined SNVs from both genes, offsetting each other’s effects (eTable 10 in the Supplement).

### IPH Progression Correlates With Messenger RNA Expression in SNVs

**Figure 2.**
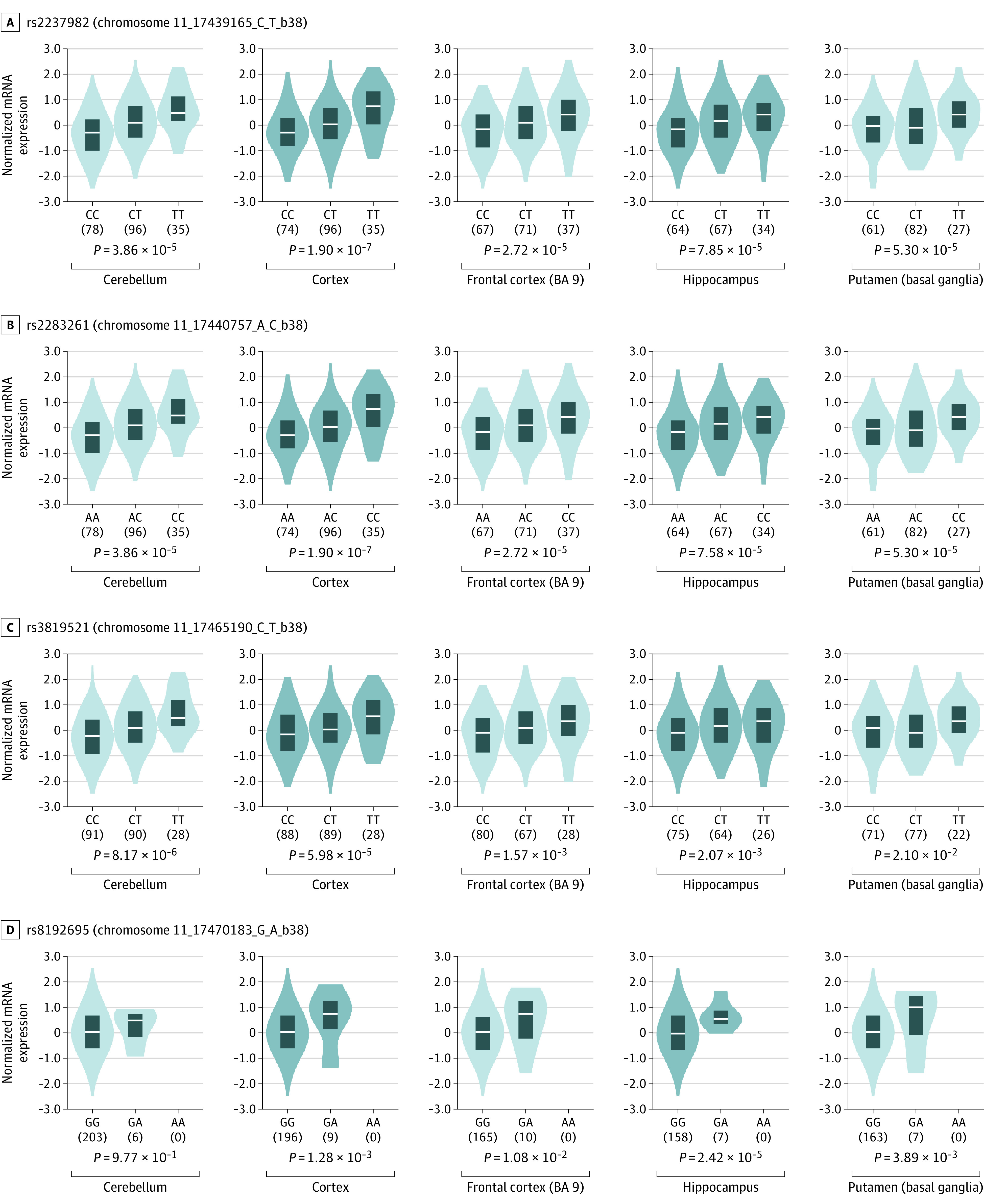
*ABCC8* Single-Nucleotide Variants (SNVs) Associated With Increased Intraparenchymal Hemorrhage (IPH) Progression in Traumatic Brain Injury (TBI) Violin plots from Genotype Tissue Expression (GTEx) portal of normalized messenger RNA (mRNA) expression levels associated with genotypes of 4 *ABCC8* SNVs associated with IPH progression. Shaded regions indicate density distribution of mRNA expression (white bar, median value). The *P* value for each SNV at each location indicates the value for different expression levels across genotypes for that SNV in the respective tissue location. The m-value denotes the posterior probability that an expression quantitative trait loci (eQTL) effect exists for this tissue based on cross-tissue meta-analyses in the GTEx project (range, 0 to 1; values closer to 1 indicate that the tissue is predicted to have a true eQTL effect). All 4 *ABCC8* SNVs are brain-specific eQTL; the 3 intronic SNVs (rs2237982, rs2283261, and rs3819521) are brain-specific eQTL in multiple tissue locations, with m-values close to or equal to 1. In all cases, mRNA expression is higher with SNVs, with a dose-dependent effect.

Only 467 of 22 922 reported *ABCC8* SNVs (2.04%) are associated with altered brain tissue messenger RNA (mRNA) levels, that is, expression quantitative trait loci (eQTL) identified by GTEx. All 4 *ABCC8* SNVs are brain-specific *cis*-eQTL (ie, alter *ABCC8* expression) (Figure 1). *ABCC8* SNVs were associated with increased IPH progression and with increased brain tissue *ABCC8* (*SUR1*) mRNA levels (Figure 2). The pathobiological role of SUR1 in regulating blood-brain barrier integrity and IPH progression suggests validity beyond statistical associations. Most brain-specific *ABCC8* eQTL cluster upstream (Figure 1). This contrasts with pancreatic *ABCC8*, in which SUR1 regulates insulin via a different channel (SUR1-K_ir_6.2): no *ABCC8* SNVs are pancreatic *cis*-eQTL, and the few *trans*-eQTL (influencing non-*ABCC8* genes) cluster downstream (Figure 1). Seventy-seven of 16 800 *TRPM4* SNVs (0.46%) are brain-specific eQTL.^26^ All 4 identified *TRPM4* SNVs are brain-specific *cis*-eQTL (eFigure 2 in the Supplement). Unlike *ABCC8*, *TRPM4* SNVs were associated with decreased IPH progression and have been conversely associated with decreased brain tissue *TRPM4* mRNA expression, particularly cerebellar and cortical (eFigure 3 in the Supplement). Brain-specific *TRPM4* eQTL cluster upstream of exon 12.

### Genotypes Improve Clinical Models of IPH Progression and Outcome

**Figure 3.**
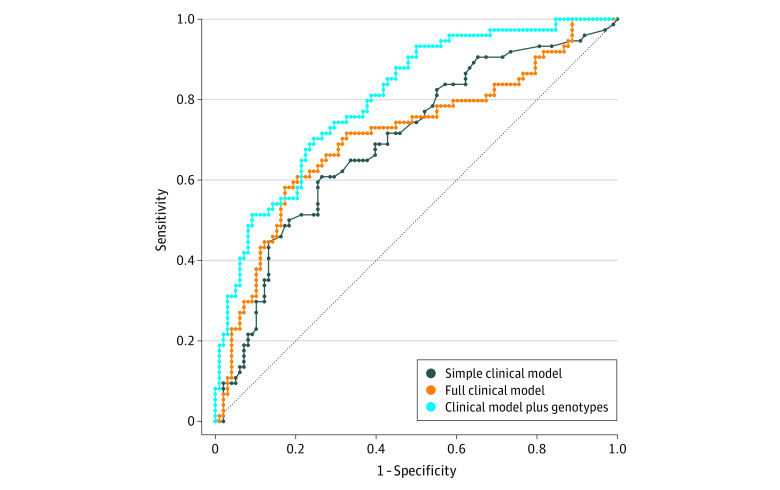
Model of Hemorrhage Progression Including *ABCC8* and *TRPM4* Genotypes vs Standard Clinical Models Receiver operating characteristic curves for different multivariable models of intraparenchymal hemorrhage (IPH) progression after severe traumatic brain injury (TBI). The simple clinical model consisting of clinical variables significantly associated with IPH progression in this cohort in a backward elimination model (ie, age and initial hemorrhage volume) provides fair discrimination with an area under the curve (AUC) of 0.6959. This is only marginally improved in the full clinical model to 0.7100 by the addition of other clinical characteristics (sex, Glasgow Coma Scale score, Injury Severity Score, partial thromboplastin time, international normalized ratio, and thrombocytopenia). The simple and full clinical models did not perform differently (*P* = .26). Addition of *ABCC8* single-nucleotide variants (SNVs) rs2237982 and rs8192695 and *TRPM4* SNVs rs909010 and rs10410857 to the basic model markedly improved model performance with an AUC of 0.8030, approaching excellent discrimination that was superior to both the simple (*P* = .004) and full (*P* = .003) models. The remaining significant SNVs were excluded from the model owing to complete overlap in patients with homozygous variants resulting in model overdetermination.

Intraparenchymal hemorrhage progression models containing clinical covariates were outperformed by adding significant *ABCC8* and *TRPM4* genotypes (Figure 3). The area under the curve for a simple clinical model containing covariates associated with progression was 0.6959. A full model (all covariates) marginally increased this to 0.7100 (Figure 3) (*P* = .26 vs simple model). Adding *ABCC8* and *TRPM4* genotypes improved the AUC to 0.8030, outperforming both full (*P* = .003) and simple (*P* = .004) clinical models. Three of the 4 *ABCC8* risk-SNVs for IPH (rs2237982, rs2283261, and rs3819521) were also associated with unfavorable 6-month Glasgow Outcome Scale scores, with significant improvement vs clinical models alone (eTable 11 in the Supplement). In simulated examples of patient selection for trials evaluating treatment effects on IPH progression, incorporating *ABCC8* and/or *TRPM4* genotypes resulted in enriched at-risk cohorts that reduced sample sizes by as much as 4-fold to detect a 30% relative risk reduction with 90% power, although potentially at the cost of a larger number of patients screened (eTable 12 in the Supplement). Selecting patients with either an *ABCC8* (risk) SNV or wild-type *TRPM4* (risk) SNV reduced the sample by 25% without increasing the number screened. Selecting patients with an *ABCC8* (risk) SNV and wild-type *TRPM4* (risk) SNV reduced the sample by approximately 76%; however, the number of patients screened doubled. Adding genotypes to a clinical model reduced sample size by approximately 50% vs all comers, marginally increasing the number screened by 20. This could affect trial efficiency, cost, and feasibility.

### Functional Implications

**Figure 4.**
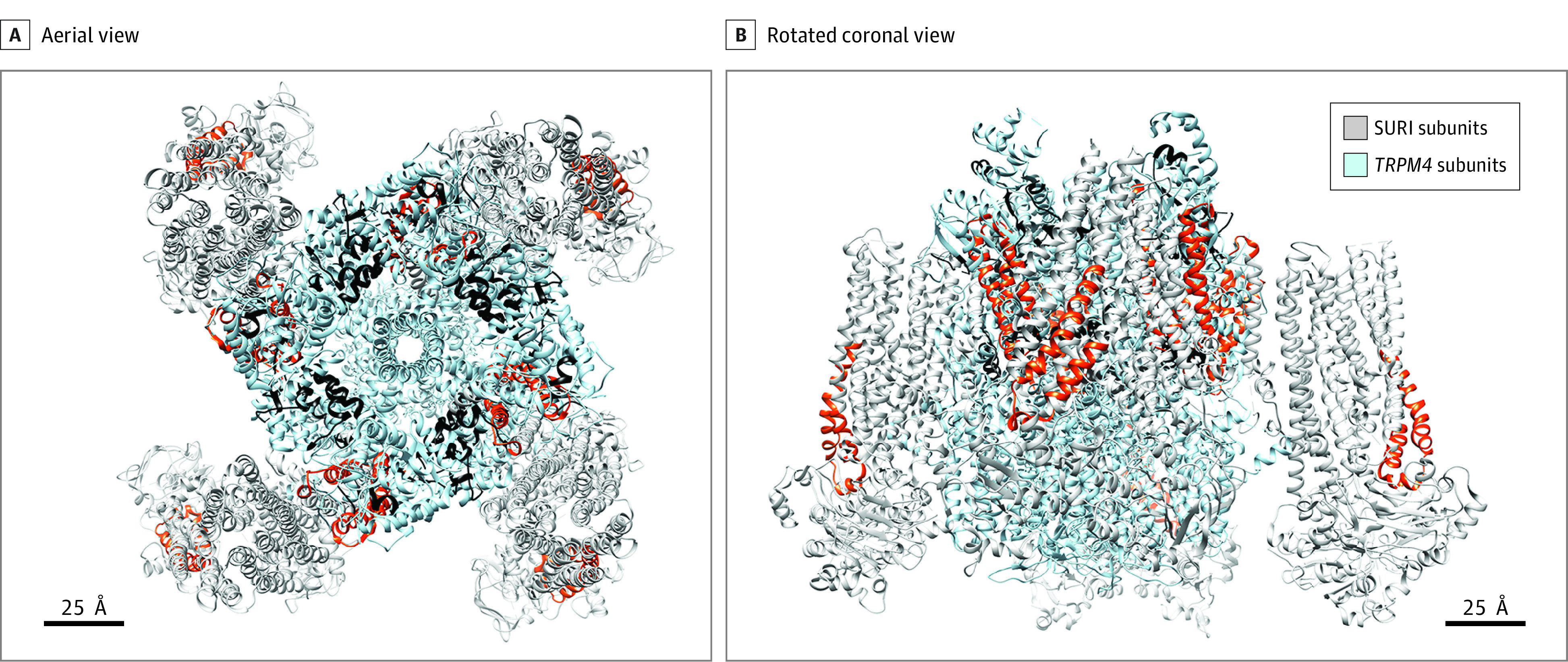
Three-Dimensional Structure of Sulfonylurea Receptor 1–Transient Receptor Potential Melastatin 4 (SUR1-TRPM4) Pore-Forming Octameric Cation Channel Involved in Hemorrhage Progression After Traumatic Brain Injury (TBI) A 3-dimensional model of the octameric structure of 4 SUR1 subunits (gray) that assemble with 4 *TRPM4* subunits (blue) to create a pore-forming nonselective cation channel. The structures were obtained from the Research Collaboratory for Structural Bioinformatics Protein Data Bank based on work by Li et al^39^ on the pancreatic channel SUR1-K_ir_6.2 and Autzen et al^40^ on the human *TRPM4* channel. University of California, San Francisco, Chimera software was used to generate the 3-dimensional structure of SUR1 in this figure without the associated K_ir_6.2 channel, which was replaced with *TRPM4* in the model. A, Aerial view illustrating the 4 SUR1 subunits binding with the 4 inner *TRPM4* subunits. Amino acid sequences encoded by regions of DNA in linkage disequilibrium (LD) with the *ABCC8* single-nucleotide variants (SNVs) associated with hemorrhage progression including exon-3 (which contains rs8192695) are highlighted in red. These sequences correspond to the sulfonylurea receptor site, as well as motifs that interface with *TRPM4*. Amino acid sequences encoded by regions of DNA in LD with *TRPM4* SNVs associated with hemorrhage progression are highlighted in black and translate to motifs that interface with SUR1. B, Aerial view rotated 90° around the x-axis to provide a coronal view.

Regulatory annotations are summarized in eTable 13 in the Supplement. Significant SNVs were in regions that influenced protein binding or transcription-factor binding with altered regulatory motifs (eTable 13 and eFigure 4 in the Supplement). Most *ABCC8* but not *TRPM4* SNVs were located in genomic regions with promoter histone and deoxyribonuclease marks. Enhancer marks were noted in three-quarters of both *ABCC8* and *TRPM4* SNVs. All *ABCC8* SNVs were in active transcription sites in brain tissue vs repressed or quiescent heterochromatin in other tissues (eFigure 5 in the Supplement). This tissue distinction was not notable for *TRPM4*. In *ABCC8*, introns 3 and 10 (housing significant SNVs) separate exons with residue-overlapping splice sites in which nucleotides of translated codons are separated in flanking exons. Intermediary intronic SNVs may thus also affect splicing. eFigure 6 in the Supplement shows linkage disequilibrium maps. A 3-dimensional model demonstrates that flanking exons and coding sequences in linkage disequilibrium with significant SNVs in both genes are translated into protein domains that constitute SUR1-TRPM4 subunit binding interfaces and the sulfonylurea receptor site/motif (Figure 4).^39,40^ The variant rs2237981 (in perfect linkage disequilibrium with rs2237982) has been associated with responsiveness to gliclazide, a selective SUR1 antagonist.^41^

## Discussion

Eight *ABCC8* and *TRPM4* SNVs were associated with IPH progression after severe TBI, significantly improving the performance of standard clinical models. *ABCC8* SNVs that were associated with increased IPH progression (Table 1 and eTable 14 in the Supplement) were also associated with increased brain tissue *ABCC8* mRNA expression, adding biological plausibility. These SNVs are located in regions containing markers of enhancers, promoters, and active transcription sites in brain tissue. *TRPM4* SNVs were associated with decreased IPH progression in our regression analysis and were associated with decreased *TRPM4* brain tissue mRNA levels based on our interrogation of the GTEx portal. The directional consistency and convergence of IPH progression and mRNA levels in both genes lend credence to these results and are congruent with known pathobiological mechanisms. Although divergent expression levels may partly underly the opposite associations of *ABCC8* vs *TRPM4* variants on IPH progression, other mechanisms may contribute, such as an effect on octameric channel assembly and efficiency, function, spliced isoforms, or sulfonylurea sensitivity. Identifying patients with risk-altering SNVs may valuably guide risk stratification, prognostication, patient selection, and clinical trial design.

### TBI IPH Progression

Variability of TBI outcome remains largely unexplained, limiting therapeutic advances. Primary injury and nonmodifiable demographic and clinical factors account for approximately 33% of outcome variability in large cohort models such as IMPACT (International Mission for Prognosis and Analysis of Clinical Trials in TBI).^42,43^ This improves only slightly after including imaging, laboratory values, and additional insults.^42^ Secondary injuries may therefore drive more than 50% of outcome variability.^2,12,24,33,42,44,45^ These processes represent substantial and likely modifiable contributors to TBI-related disability, providing an opportunity for targeting host response. Genetic variation may modify host response/secondary injury and thereby influence outcomes. Consistent with previous reports, IPH progression, a devastating form of potentially treatable secondary injury, occurred in 102 patients in our cohort and was associated with increased odds of mortality by more than 3-fold and with decreased favorable functional outcome by as much as 70% (eTable 5 in the Supplement). No therapy targeting IPH progression has yet demonstrated benefit in severe TBI, including tranexamic acid.^46,47,48^ An urgent need for translatable, targeted treatments remains.

### SUR1-TRPM4 and Hemorrhage Progression

SUR1 has historically been studied in pancreatic glucose metabolism, where it regulates K_ir_6.2. Its role in the central nervous system has been increasingly appreciated since the discovery of SUR1-TRPM4.^2,49,50^ De novo channel upregulation after injury provides a unique opportunity for early molecularly directed intervention with minimal adverse effects. Results from randomized trials (eg, CHARM^13^ and ASTRAL^14^) are eagerly awaited. However, given disappointing results from multiple prior TBI trials, a precision medicine–based approach might valuably inform and advance effective translation of this therapy. Appropriate patient selection, a priori determination of high-risk subgroup analyses, and identification of likely treatment responders could guide and improve trial design. Genetic differences may contribute to variation in SUR1-TRPM4 regulation, expression, posttranslational modification, subunit interaction, or channel function, thereby affecting individuals’ risks of IPH progression and whether (or how) they respond to targeted treatments. Understanding the spatial clustering of SNVs associated with secondary injury may also facilitate identification of specific regions in the SUR1-TRPM4 complex that may be fruitful targets for future drug development.

### *ABCC8* and *TRPM4* Sequence Variations in TBI

Most *ABCC8* SNVs reported in glucose metabolism disorders are downstream, proximal to KCNJ11 (K_ir_6.2).^6^ None are pancreatic *cis*-eQTL. In contrast, *ABCC8* risk SNVs in TBI are all upstream eQTL, where most brain-specific *ABCC8* eQTL are located (Figure 1). Three *ABCC8* SNVs associated with IPH progression (rs2237982, rs2283261, and rs3819521) are independently associated with intracranial hypertension in previous work in this cohort.^5,6,24^ Single-nucleotide variants were associated with increased odds of both intracranial hypertension and IPH progression. Given the association among intracranial pressure, cerebral edema, and the continuum with IPH progression, these findings are reassuring and physiologically consistent. These high-risk SNVs are also associated with increased brain tissue *ABCC8* mRNA levels (Figure 2). Regulatory annotations demonstrate that significant *ABCC8* SNVs are located in gene regions with strong promoter marks, enhancer marks, and active transcription sites in the brain vs quiescent heterochromatin regions in other tissues (eTable 13 and eFigures 4 and 5 in the Supplement). This may underpin the increased mRNA levels. Although increased mRNA does not necessarily dictate increased protein expression, it is directionally consistent and provides convergent validity and biological support to the identified association. Based on the underlying pathobiology, increased SUR1 expression may facilitate cerebral edema and IPH progression.

Spatial clustering of significant sequence variants occurred around critical regions of DNA interspersed between sequences encoding protein domains that constituted the SUR1-TRPM4 subunit binding interface and the sulfonylurea receptor motif. These may contain variants that could differentially affect octameric channel assembly and efficiency, function, or sulfonylurea sensitivity, such as rs2237981. Single-nucleotide variants within residue-overlapping splice sites may affect splicing and SUR1 isoforms. Fewer brain-specific enhancer marks and active transcription sites were evident in genomic regions containing the *TRPM4* SNVs, consistent with lower mRNA expression. True functional consequences of the significant SNVs remain unknown, and future evaluation of biological causality is essential. Given the demonstrated involvement of SUR1-TRPM4 in several acute neurological diseases, understanding the effects of these variants may have implications beyond TBI.

### Precision Medicine for TBI

Point-of-care genotyping is emerging for several diseases.^51,52^ Genetic data are increasingly used across medical specialties to inform clinical practice by classifying disease subtypes, treatment responders, and risk stratification.^21,51,53,54^ Identifying *ABCC8* and *TRPM4* genotypes that influence IPH progression could be a valuable clinical and research tool. Early knowledge of genotypes may help risk stratify and prognosticate at presentation. Determining functional consequences of these SNVs in biological models may reveal causal mechanisms of secondary injury, facilitating development of novel gene-based and targeted therapy. Arguably, one of the most exciting and relevant possibilities of identifying high- vs low-risk *ABCC8/TRPM4* variants is to enrich patient selection, inform subgroup analysis, and guide clinical trial design. If validated, our results suggest that enriched genotype-based patient selection could reduce sample size and trial cost while improving efficiency and feasibility. This will facilitate selection of those likely to benefit, because low-risk patients may dilute detectable effects of therapy. Ultimately, genotype profiling may inform or identify treatment response. Despite promising results from preclinical and clinical studies of SUR1-TRPM4 inhibition, it has yet to be tested and demonstrate benefit in large randomized clinical trials. Numerous previous therapies in TBI at this juncture have ultimately failed to translate. Complementing neuroimaging and endophenotyping, multimodal monitoring, and biomarkers with targeted genetic information to optimize trial design and patient management may advance the future of TBI therapeutics.

### Limitations

This study has some limitations. Our cohort is small for genetic studies. Nonetheless, it is one of the largest with available genetic information in severe TBI, and, to our knowledge, the largest evaluating association with IPH progression. One other study has identified *APOE-ε4* as being associated with progression in 123 patients.^22^ This was a candidate-gene study, thereby decreasing sample size requirements and significance thresholds vs genome-wide approaches. Multicenter collaborations such as TRACK-TBI (Transforming Research and Clinical Knowledge in Traumatic Brain Injury) with 18 enrolling sites have approximately 300 patients with severe TBI, many overlapping with our cohort. Recent TRACK-TBI publications reporting important genetic contributions have pilot cohorts of 93 to 220 patients (including those with mild-to-moderate TBI).^7,55,56^ An ongoing transatlantic initiative, GAIN (Genetic Associations in Neurotrauma), is combining samples from TRACK-TBI and its European corollary (CENTER-TBI [Collaborative European NeuroTrauma Effectiveness Research in TBI]). This endeavor, although unavoidably protracted and resource intensive, is essential to validate single-center data and propel precision medicine–based TBI care to a clinical reality.

We observed large, clinically meaningful effects that retained statistical significance after adjusting for multiple comparisons, despite conservative definitions of IPH progression. Although this reduces the likelihood of false-positive associations, it may underestimate significant SNVs. Our cohort was limited to severe TBI. Female patients (23.1%) and non-White patients (8.4%) were underrepresented. Genetic variation may differentially affect IPH progression in these subgroups. Tertiary care center enrollment is prone to selection bias. We focused on *ABCC8* (regulatory protein) and *TRPM4* (pore-forming subunit); however, several related genes in this pathway may affect host response. Confounding by population stratification is an important limitation of allelic association and/or case-control studies. Although principal components analysis in this cohort does not identify meaningful stratification, it remains a possibility. In addition, sample homogeneity may limit generalizability. Further work addressing these limitations is important and facilitated by multicenter collaborations such as GAIN. Although this cohort contains approximately 5000 patients, only approximately 10% have severe TBI. Nonetheless, this represents as close to ideal a cohort as currently possible for replication.

## Conclusions

In a cohort of patients with severe TBI included in this genetic association study, 8 *ABCC8* and *TRPM4* SNVs were associated with IPH progression in the first 5 days after injury. Spatial clustering, brain-specific eQTL, and regulatory annotations suggest biological plausibility. Genetic influences on this critical secondary injury may have important implications for risk stratification, patient selection, and precision medicine, including trial design for SUR1-TRPM4 inhibition, and may also inform drug development targeting the SUR1-TRPM4 complex.

## Notes

Supplement. **eMethods.** Outcomes, Measures, and Analysis **eTable 1.** List of All Genotyped SNPs in *ABCC8* and *TRPM4* **eTable 2.** Cohort Demographics, Clinical Characteristics and Association With Hemorrhage Progression in TBI **eTable 3.** Clinical Characteristics and Outcomes of All Patients With Severe TBI During Enrollment Period **eTable 4.** Cohort Demographics and Clinical Characteristics of Progressors and Nonprogressors **eTable 5.** Association of Intraparenchymal Hemorrhage Progression With Outcome After Severe TBI **eTable 6.** Genotype Frequencies of *ABCC8* and *TRPM4* SNPs Associated With Hemorrhage Progression in TBI **eTable 7.** *ABCC8* and *TRPM4* Single-Nucleotide Polymorphisms Associated With Quantitative Change in Intraparenchymal Hemorrhage Volumes in Severe TBI **eTable 8.** *ABCC8* and *TRPM4* Single-Nucleotide Polymorphisms Associated With Intraparenchymal Hemorrhage Progression in Severe TBI (Full Cohort) **eTable 9.** *ABCC8* and *TRPM4* Haplotypes Associated With Contusion Expansion in Severe TBI **eTable 10.** Haplotype Distribution in the Cohort of Severe TBI **eTable 11.** Intraparenchymal Hemorrhage Progression Risk Polymorphisms Associated With 6-mo Glasgow Outcome Scale (GOS) Score **eTable 12.** Sample Size Calculations for Genotype-Based Patient Selection in Clinical Trial **eTable 13.** Regulatory Annotations of *ABCC8* and *TRPM4* Single-Nucleotide Polymorphisms Associated With Hemorrhage Progression in Severe TBI **eTable 14.** Association of *ABCC8* and *TRPM4* Single-Nucleotide Polymorphisms With IPH Progression (Quantitative Definition Only) **eFigure 1.** Opposite Associations of *ABCC8* vs *TRPM4* Variant SNPs With Intraparenchymal Hemorrhage Progression in TBI **eFigure 2.** Spatial Distribution of *TRPM4* SNPs in Hemorrhage Progression After TBI **eFigure 3.** Variant *TRPM4* SNPs Associated With Decreased Hemorrhage Progression in TBI Are eQTL With Decreased *TRPM4* Expression in Cerebellum **eFigure 4.** Sequence Logos Demonstrating Impact of *ABCC8* and *TRPM4* SNPs on Transcription Factor Motifs **eFigure 5.** Brain Chromatin States of *ABCC8* and *TRPM4* Genomic Loci **eFigure 6.** Linkage Disequilibrium Maps for *ABCC8* and *TRPM4* SNPs Regional Loci eReferences
