# Supplementary material for: Genetic Variants Associated With Intraparenchymal Hemorrhage Progression After Traumatic Brain Injury
Source: JAMA Netw Open. 2021 Jul 26;4(7):e2116839. doi: 10.1001/jamanetworkopen.2021.16839 (PMC8314141; doi:10.1001/jamanetworkopen.2021.16839)
Supplement: Supplement. — eMethods. Outcomes, Measures, and Analysis eTable 1. List of All Genotyped SNPs in ABCC8 and TRPM4 eTable 2. Cohort Demographics, Clinical Characteristics and Association With Hemorrhage Progression in TBI eTable 3. Clinical Characteristics and Outcomes of All Patients With Severe TBI During Enrollment Period eTable 4. Cohort Demographics and Clinical Characteristics of Progressors and Nonprogressors eTable 5. Association of Intraparenchymal Hemorrhage Progression With Outcome After Severe TBI eTable 6. Genotype Frequencies of ABCC8 and TRPM4 SNPs Associated With Hemorrhage Progression in TBI eTable 7. ABCC8 and TRPM4 Single-Nucleotide Polymorphisms Associated With Quantitative Change in Intraparenchymal Hemorrhage Volumes in Severe TBI eTable 8. ABCC8 and TRPM4 Single-Nucleotide Polymorphisms Associated With Intraparenchymal Hemorrhage Progression in Severe TBI (Full Cohort) eTable 9. ABCC8 and TRPM4 Haplotypes Associated With Contusion Expansion in Severe TBI eTable 10. Haplotype Distribution in the Cohort of Severe TBI eTable 11. Intraparenchymal Hemorrhage Progression Risk Polymorphisms Associated With 6-mo Glasgow Outcome Scale (GOS) Score eTable 12. Sample Size Calculations for Genotype-Based Patient Selection in Clinical Trial eTable 13. Regulatory Annotations of ABCC8 and TRPM4 Single-Nucleotide Polymorphisms Associated With Hemorrhage Progression in Severe TBI eTable 14. Association of ABCC8 and TRPM4 Single-Nucleotide Polymorphisms With IPH Progression (Quantitative Definition Only) eFigure 1. Opposite Associations of ABCC8 vs TRPM4 Variant SNPs With Intraparenchymal Hemorrhage Progression in TBI eFigure 2. Spatial Distribution of TRPM4 SNPs in Hemorrhage Progression After TBI eFigure 3. Variant TRPM4 SNPs Associated With Decreased Hemorrhage Progression in TBI Are eQTL With Decreased TRPM4 Expression in Cerebellum eFigure 4. Sequence Logos Demonstrating Impact of ABCC8 and TRPM4 SNPs on Transcription Factor Motifs eFigure 5. Brain Chromatin States of ABCC [file jamanetwopen-e2116839-s001.pdf]

Supplementary Online Content

Jha RM, Zusman BE, Puccio AM, et al. Genetic variants associated with intraparenchymal hemorrhage progression after traumatic brain injury. *JAMA Netw Open*. 2021;4(7):e2116839. doi:10.1001/jamanetworkopen.2021.16839

eMethods. Outcomes, Measures, and Analysis

eTable 1. List of All Genotyped SNPs in *ABCC8* and *TRPM4*

eTable 2. Cohort Demographics, Clinical Characteristics and Association With Hemorrhage Progression in TBI

eTable 3. Clinical Characteristics and Outcomes of All Patients With Severe TBI During Enrollment Period

eTable 4. Cohort Demographics and Clinical Characteristics of Progressors and Nonprogressors

eTable 5. Association of Intraparenchymal Hemorrhage Progression With Outcome After Severe TBI

eTable 6. Genotype Frequencies of *ABCC8* and *TRPM4* SNPs Associated With Hemorrhage Progression in TBI

eTable 7. *ABCC8* and *TRPM4* Single-Nucleotide Polymorphisms Associated With Quantitative Change in Intraparenchymal Hemorrhage Volumes in Severe TBI

eTable 8. *ABCC8* and *TRPM4* Single-Nucleotide Polymorphisms Associated With Intraparenchymal Hemorrhage Progression in Severe TBI (Full Cohort)

eTable 9. *ABCC8* and *TRPM4* Haplotypes Associated With Contusion Expansion in Severe TBI

eTable 10. Haplotype Distribution in the Cohort of Severe TBI

eTable 11. Intraparenchymal Hemorrhage Progression Risk Polymorphisms Associated With 6-mo Glasgow Outcome Scale (GOS) Score

eTable 12. Sample Size Calculations for Genotype-Based Patient Selection in Clinical Trial

eTable 13. Regulatory Annotations of *ABCC8* and *TRPM4* Single-Nucleotide Polymorphisms Associated With Hemorrhage Progression in Severe TBI

eTable 14. Association of *ABCC8* and *TRPM4* Single-Nucleotide Polymorphisms With IPH Progression (Quantitative Definition Only)

eFigure 1. Opposite Associations of *ABCC8* vs *TRPM4* variant SNPs with Intraparenchymal Hemorrhage Progression in TBI

eFigure 2. Spatial Distribution of *TRPM4* SNPs in Hemorrhage Progression After TBI

eFigure 3. Variant *TRPM4* SNPs Associated With Decreased Hemorrhage Progression in TBI are eQTL With Decreased *TRPM4* Expression in Cerebellum

eFigure 4. Sequence Logos Demonstrating Impact of *ABCC8* and *TRPM4* SNPs on Transcription Factor Motifs

eFigure 5. Brain Chromatin States of *ABCC8* and *TRPM4* Genomic Loci

eFigure 6. Linkage Disequilibrium Maps for *ABCC8* and *TRPM4* SNPs Regional Loci

eReferences

© 2021 Jha RM et al. *JAMA Network Open*.

This supplementary material has been provided by the authors to give readers additional information about their work.

© 2021 Jha RM et al. *JAMA Network Open*.

eMethods. Outcomes, Measures, and Analysis

DNA collection and Genotyping

DNA was extracted and genotyped per established methods[1–4]. Briefly, *ABCC8* and *TRPM4* exonic SNPs were genotyped using Illumina (Human-Core Exome v1.0) multiplex platform by the Center for Inherited Disease Research. Selection was unbiased and included all *ABCC8* and *TRPM4* SNPs covered by the chip (n=25). For *ABCC8* intron coverage, 15 tag-SNPs were identified using HapMap with the tagger-algorithm pairwise approach, $r^2 \geq 0.8$ and minor allele frequency >0.20 and genotyped using iPlex-Gold with an Agena Compact MassArray with Nanodispenser. Genotype analysis was performed using Typer-4.0. Assay. 40 SNPs were interrogated (Supplemental-Table 1). Genotyping researchers were blinded to demographic and outcome data. Data cleaning and quality control included blind technical duplicates, Hardy-Weinberg Equilibrium (HWE) testing, and excluding SNPs that did not have a minimum 95% call-rate. Principal-component analysis used for ancestry filtering assessed for population stratification and identified a single cluster, with the small numbers outside of that cluster being too few to analyze.

Hemorrhage Progression

Serial CT scans were assessed for intraparenchymal hemorrhage progression at presentation, 6h, 24h and 120h. Hemorrhage progression was determined using two criteria- quantitative estimation of traumatic intraparenchymal hemorrhage (IPH) volumes, and official neuroradiologist interpretations. Both criteria had to be fulfilled for a scan to qualify as demonstrating progression. We selected this two-part criteria to identify hemorrhage progression to prioritize the specificity rather than the sensitivity of the definition. This conservative approach was used to minimize the likelihood of false positive associations.

- Hemorrhage volume quantification: total IPH volumes were estimated using the standard ABC/2 calculation[5, 6]. Based on previous literature, hemorrhage progression was defined as an increase of 6 mL in ICH volume from admission CT, an increase of IPH volume by 30% from admission CT, or the appearance of a new intra-axial hemorrhage from the admission CT[6]. First appearance of catheter-tract hemorrhages were not counted towards progression, however subsequent enlargement of catheter tract hemorrhages qualified for inclusion in volumetric calculations. 10% of all CTs were independently reviewed in a blinded fashion, and yielded excellent interclass correlation (0.917).
- Neuroradiologist interpretation: All final neuroradiologist reports were manually read by trained research staff blind to patient genotype for phrasing indicating progression of the hemorrhagic component of the

© 2021 Jha RM et al. *JAMA Network Open*.

IPH specifically. Reports were searched for the terms including “expanded”, “blossomed”, “increased” or “new” in reference to the hemorrhagic component of the IPH only while terms such as “evolved” or “matured” were felt to be more representative of the natural course of disease rather than progression. To minimize likelihood of false positive results, reports where expansion was not mentioned were interpreted as stability.

- Comparison of the two independent metrics of hemorrhage progression yielded a percent agreement of 88.9% and a Cohen’s kappa of 0.78, suggesting substantial agreement between the two metrics.

SNP Functional Potential Determination

SNPs were evaluated for impact on gene expression using the Genotype Tissue Expression (GTEx) Project data portal (www.gtexportal.org, 06/23/2020) [7]. They were interrogated for brain-specific gene expression quantitative trait loci (eQTL) in the hippocampus, non-specified cortex, frontal cortex, putamen, and cerebellum to assess breadth and consistency of impact on gene expression across cortical vs. deep brain structures. Regulatory potential was evaluated using RegulomeDB v2.0 and HaploReg V4.1[8–10]. SNPs loci were explored for the ~200 bp regional chromatin state, transcription start sites, promoter histone marks, enhancer histone marks, DNAse, and protein binding (via Chromatin-ImmunoPrecipitation, ChIP, reports) in brain vs. all reported tissues. Individual SNPs were interrogated for impact on altering regulatory motifs for transcription factors using sequence logos (RegulomeDB) as well as position-weighted matrix (PWM) scores (HaploReg). PWM scores on Haploreg (determined using experimental data on JASAPR, TRANSFAC, and protein binding microarray experiments) are available as log-odds, and account for motif lengths and base-pair compositions; they reflect transcription factor binding affinity[9]. Log-odds score differences between variant allele vs reference alleles evaluate change in binding affinity[9] (positive values reflect an increase in log-odds score for variant alleles, and suggests increased transcription factor binding strength). SNPs were evaluated for reported clinical significance via systematic PubMed, Embase, and ClinVar searches.

Spatial relationship modeling between *ABCC8* and *TRPM4* loci and channel structure

Chromosomal locations were identified using the University of California, Santa Cruz genome browser, human-genome assembly(hg-38). Linkage disequilibrium (LD), distance from the proximal exon, peptide sequences encoded by specific exons and residue overlap splice sites were identified via Ensembl-100[11]. Established SUR1 (5WUA) and TRPM4 (6BQV) 3-dimensional electron microscopy structures were obtained from the Research Collaboratory for Structural Bioinformatics Protein Data Bank[12–15]. University of California, San Francisco Chimera was used to generate the octameric SUR1-TRPM4 channel[16].

© 2021 Jha RM et al. *JAMA Network Open*.

Statistical Analysis: Sample Size Impact Simulation

We also simulated an example to demonstrate the potential impact of *a priori* knowledge of patients' *ABCC8* and/or *TRPM4* genotypes on patient selection for clinical-trial design. We stratified our sample by genotypes, and, based on the genotype-subgroup, looked at both the sample size required and the number of patients who would need to be genotyped in order to have a 90% power to find a 30% relative risk reduction (RRR) in the risk of intra-axial hemorrhage progression within 120 hours. For this, we first calculated the probability of belonging to a specific genotype subgroup and the probability of hemorrhage progression within that subgroup for each of the following categories: 1) all comers (current trial designs), 2) at least one *ABCC8* SNP with homozygous variant (risk) genotype, 3) at least one *TRPM4* SNP with homozygous wildtype (risk) genotype, 4) at least one *ABCC8* SNP with homozygous variant (risk) phenotype AND at least one *TRPM4* SNP with homozygous wildtype (risk) genotype, 5) and at least one *ABCC8* SNP with homozygous variant (risk) phenotype OR at least one *TRPM4* SNP with homozygous wildtype (risk) genotype, 6) patients predicted to have a >50% probability of hemorrhage progression by the full clinical model without genotypes used for creating the ROC curves, 7) and patients predicted to have a >50% probability of hemorrhage progression by the full clinical plus genotypes model used for creating the ROC curves. The probability of hemorrhage progression for each subgroup was then used in sample size calculations to design different iterations of the hypothetical study where a treatment is expected to result in a RRR of hemorrhage progression by 30% with a power of 0.9. The obtained sample size was divided by the proportion of patients within that subgroup to determine how many patients would need to be genotyped to reach the necessary enrollment sample size. This procedure was followed for all 5 subgroups to identify the degree to which genotype-based patient selection may impact sample size based on varying risk of hemorrhage progression.

eTable 1. List of All Genotyped SNPs in *ABCC8* and *TRPM4*

Gene	SNPs genotyped
ABCC8	rs7105832, rs2237982, rs11024286, rs4148622, rs2283261, rs381952, rs2283258, rs1799857, rs8192695, rs11024273, rs2074311, rs2237991, rs2299639, rs3758953, rs4148618, rs4148641, rs4757517, rs7124355, rs7947462, rs916827, rs1109591, rs757110, rs1799859, rs4148640, rs2074312, rs2106865, rs2074315, rs4757517, rs7119439, rs1048099
TRPM4	rs8104571, rs150391806, rs11667393, rs3760666, rs113984787, rs1477363, rs10410857, rs56355369, rs909010, rs145847114

© 2021 Jha RM et al. *JAMA Network Open*.

eTable 2. Cohort Demographics, Clinical Characteristics and Association With Hemorrhage Progression in TBI				
**Characteristic**	**Distribution**		**5-day Hemorrhage Progression OR (95% CI)**	
	Entire TBI Cohort (n=416)	No-Craniectomy Primary Cohort (n=321)	Entire TBI Cohort	No-Craniectomy Primary Cohort
Age in years: mean (SD)	38.6 (16.9)	37.0 (16.3)	1.02 (1.01-1.03)*	1.03 (1.01-1.04)*
Sex: n (%)				
Male	324 (77.9)	247 (76.9)	Ref	Ref
Female	92 (22.1)	74 (23.1)	0.74 (0.44-1.23)	0.79 (0.44-1.43)
Admission GCS: median (IQR)	6 (5-7)	7 (5-7)	0.87 (0.76-1.01)	0.98 (0.83-1.15)
ISS: mean (SD)	31.8 (11.1)	32.6 (11.4)	1.0 (0.98-1.02)	1.0 (0.98-1.02)
Race: n (%)				
White	381 (91.6)	293 (91.6)	Ref	Ref
African American	30 (7.2)	23 (7.2)	0.75 (0.32-1.76)	0.76 (0.29-1.99)
Southeast Asian	4 (1)	4 (1.3)	1.25 (0.14-7.36)	1.30 (0.18-9.35)
Unknown	1 (0.2)	1 (0.3)	-[a]	
Mechanism of Injury: n (%)				
Enclosed Motor Vehicle Accident	216 (51.9%)	185 (57.6%)	Ref	Ref
Motor Cycle Crash	67 (16.1%)	58 (18.1%)	1.08 (0.60-1.94)	1.37 (0.73-2.57)
Fall	87 (20.9%)	50 (15.6%)	2.10 (1.19-3.72)*	2.04 (1.02-4.09)*
Assault	17 (4.1%)	10 (3.2%)	1.34 (0.50-3.63)	0.66 (0.16-2.64)
Gun Shot Wound	2 (0.5%)	1 (0.3%)	0.55 (0.20-1.51)	1.53 (0.09-24.98)
Other[b]	23 (5.5%)	15 (4.7%)	0.55 (0.20-1.51)	0.58 (0.15-2.25)
Unknown	4 (1%)	2 (0.6%)	3.58 (0.37-35.05)	-
Primary Injury Pattern: n (%)				
None	9 (2.5%)	9 (3.2%)	- [c]	-
Subdural Hematoma	115 (31.9%)	55 (19.8%)	Ref	Ref
Epidural Hematoma	21 (5.8%)	16 (5.8%)	0.73 (0.27-2.00)	0.76 (0.23-2.47)
Subarachnoid Hemorrhage	60 (16.6%)	55 (19.8%)	0.39 (0.19-0.81)*	0.59 (0.23-1.40)
Intraparenchymal Hemorrhage	105 (29.9%)	92 (33.1%)	0.46 (0.26-0.82)*	0.85 (0.18-1.11)
Intraventricular Hemorrhage	10 (2.8%)	10 (3.6%)	- [d]	-
Diffuse Axonal Injury	41 (11.4%)	41 (14.8%)	0.28 (0.12-0.61)*	0.45 (0.18-2.09)
Admission Platelet Count, 10^9/L: mean (SD)	228.8 (71.4)	229.9 (66.0)	1.00 (1.00-1.00)	1.00 (0.99-1.00)
Admission Thrombocytopenia <100x10^9/L: n(%)	14 (3.4)	7 (2.2)	1.46 (0.45-4.69)	1.31 (0.26-6.62)
Admission PTT, sec: mean (SD)	27.6 (5.6)	27.5 (5.7)	1.04 (0.99-1.08)	1.01 (0.97-1.06)
Admission INR: median (IQR)	1.2 (1.1-1.3)	1.1 (1.1-1.3)	1.72 (0.72-4.15)	1.22 (0.51-2.93)
Admission Anticoagulant Use, Yes: n (%)	9 (2.2%)[e]	4 (1.3%)	-[f]	-
Admission Antiplatelet Use, Yes: n (%)	28 (7.0%)	21 (6.9%)	0.89 (0.39-2.05)	0.86 (0.32-2.30)
Craniectomy, Yes: n (%)	124 (29.8%)	30 (9.4%)	5.13 (3.10-8.52)*	22.6 (5.24-97.06)
Early Craniectomy, Yes: n (%)[f]	95 (22.8)	0 (0%)	2.92 (1.72-4.96)*	-
Admission IPH Volume, mL: median (IQR)	1.1 (0.2-6.7)	1 (0.2-4.4)	1.01 (0.99-1.02)	1.03 (1.00-1.05)*
Admission IPH Volume >1.5mL, Yes: n (%)	181(43.5%)	133 (41.4%)	1.81(1.19-2.77)*	2.89 (1.76-4.75)*
Hemorrhage Progression (Yes)				
6h	80 (31.1%)	54 (27.7%)	NA	
24h	132 (43.14%)	90 (38.46%)	NA	
5d	150 (49.02%)	102 (43.6%)	NA	

CI= confidence interval; GCS= Glasgow Coma Scale Score; OR= Odds Ratio; PTT= Partial Thromboplastin Time; INR= International Normalized Ratio; IQR=inter quartile range; n=number; %= percentage; IPH=intraparenchymal hemorrhage; SD= standard deviation
*Indicates statistical significance at $p<0.05$.
[a]Only one patient had unknown race, so effect could not be estimated.
[c]Other includes: Hit by falling object (n = 6), explosion (n = 1), recreational sports (n = 1), bicycle vs vehicle (n = 7), pedestrian vs vehicle (n = 4), other not otherwise specified (n = 4).
[c]Patients with no primary injury pattern had no blood on their CT, and therefore could not have effect estimates.
[d]No patient with a primary injury pattern of intraventricular hemorrhage progressed.
[e]All patients using anticoagulants in the cohort were taking coumadin.
[e]All patients using anticoagulants in the cohort progressed.

© 2021 Jha RM et al. *JAMA Network Open*.

[f] Early craniectomy was performed in 70 patients for SDH, 8 patients for EDH, 14 patients for intraparenchymal hematoma evacuation, and 3 patients for intractable intracranial pressure.

© 2021 Jha RM et al. *JAMA Network Open*.

eTable 3. Clinical Characteristics and Outcomes of All Patients With Severe TBI During Enrollment Period		
	All Severe TBI Patients (n = 541)	Primary analysis sample (n = 321)
Age, years (mean±SD)	39.9±17.1	37.0±16.3
Sex, n (%)		
Male	424 (78.4%)	247 (76.9%)
Female	117 (21.6%)	74 (23.1%)
Admission GCS (median [IQR])	6 (4-7)	7 (5-7)
6-month GOS score (median [IQR])	3 (1-4)	3 (1-4)

SD: Standard Deviation; GCS: Glasgow Coma Scale score; GOS: Glasgow Outcome Scale score; IQR: interquartile range

© 2021 Jha RM et al. *JAMA Network Open*.

**eTable 4. Cohort Demographics and Clinical Characteristics of Progressors and Nonprogressors**			
**Characteristic**			
	No progression within 5d (n=204)	Progression within 5d (n=117)	*p-value*
Age in years: mean (SD)	33.8 (15.4)	40.6 (16.2)	<0.001[a]
Sex: n (%)			0.52
Male	153 (75.0)	94 (80.3)	
Female	51 (25.0)	23 (19.7)	
Admission GCS: median (IQR)	7 (5-7)	7 (5-7)	0.88
ISS: mean (SD)	32.6 (11.4)	32.5 (11.9)	0.97
Race: n (%)			0.82
White	186 (91.2)	107 (91.5)	
African American	16 (7.8)	7 (6.0)	
Southeast Asian	2 (1)	2 (1.7)	
Unknown	0 (0)	1 (0.9)	
Mechanism of Injury: n (%)			0.12
Enclosed Motor Vehicle Accident	126 (61.8%)	60 (51.3%)	
Motor Cycle Crash	32 (15.7%)	25 (21.4%)	
Fall	26 (12.8%)	24 (20.5%)	
Assault	7 (3.4%)	2 (1.7%)	
Gun Shot Wound	1 (0.5%)	1 (0.9%)	
Other[b]	12 (5.9%)	3 (2.6%)	
Unknown	0 (0%)	2 (1.7%)	
Primary Injury Pattern: n (%)			0.02[a]
None	9 (5.1%)	0 (0%)	
Subdural Hematoma	32 (18.1%)	23 (22.3%)	
Epidural Hematoma	9 (5.1%)	**7** (6.8%)	
Subarachnoid Hemorrhage	38 (21.5%)	17 (16.5%)	
Intraparenchymal Hemorrhage	51 (28.8%)	43 (41.8%)	
Intraventricular Hemorrhage	10 (5.7%)	0 (0%)	
Diffuse Axonal Injury	28 (15.8%)	13 (12.6%)	
Admission Platelet Count, 10^9/L: mean (SD)	231.2 (62.2)	225.7 (71.2)	0.49
Admission Thrombocytopenia <100x10^9/L: n(%)	4 (2.0)	2 (1.7)	0.62
Admission PTT, sec: mean (SD)	27.2 (6.6)	27.5 (4.4)	0.69
Admission INR: median (IQR)	1.1 (1.1-1.2)	1.1 (1.1-1.3)	0.94
Admission Anticoagulant Use, Yes: n (%)	0 (0.0%)[e]	3 (2.6%)	0.08
Admission Antiplatelet Use, Yes: n (%)	14 (6.8%)	7 (6.5%)	0.77
Admission IPH Volume, mL: median (IQR)	0.8 (0.3-2.8)	2.9 (0.7-9.5)	<0.001[a]
Admission IPH Volume >1.5mL, Yes: n (%)	60 (29.7%)	72 (61.5%)	<0.001[a]

GCS= Glasgow Coma Scale Score; OR= Odds Ratio; PTT= Partial Thromboplastin Time; INR= International Normalized Ratio; IQR=inter quartile range; n=number; %= percentage; IPH=intraparenchymal hemorrhage; SD= standard deviation

[a]Indicates statistical significance at *p<0.05*

[b]Other includes: Hit by falling object, explosion, recreational sports, bicycle vs vehicle, pedestrian vs vehicle , other not otherwise specified.

[c]Patients with no primary injury pattern had no blood on their CT, and therefore could not have effect estimates.

[d]No patient with a primary injury pattern of intraventricular hemorrhage progressed.

[e]All patients using anticoagulants in the cohort were taking coumadin.

© 2021 Jha RM et al. *JAMA Network Open*.

eTable 5. Association of Intraparenchymal Hemorrhage Progression With Outcome After Severe TBI												
	**Univariable associations, Odds Ratio (95% CI)**						**Multivariable[a] Association Odds Ratio (95% CI)**					
	**6h**	***p*-value**	**24h**	***p*-value**	**120h**	***p*-value**	**6h**	***p*-value**	**24h**	***p*-value**	**120h**	***p*-value**
**Discharge Mortality**	3.37 (1.78-6.38)	<0.001	2.57 (1.44-4.57)	0.001	3.31 (1.80-6.08)	<0.001	1.98 (0.92-4.27)	0.08	1.58 (0.81-3.10)	0.18	2.27 (1.14-4.55)	0.02
**Favorable GOS (≥4)**												
**3 months**	0.41 (0.20-0.84)	0.02	0.45 (0.24-0.82)	0.01	0.48 (0.27-0.87)	0.02	0.35 (0.15-0.83)	0.02	0.42 (0.20-0.85)	0.02	0.48 (0.24-0.96)	0.04
**6 months**	0.31 (0.16-0.59)	<0.001	0.31 (0.18-0.54)	<0.001	0.30 (0.17-0.51)	<0.001	0.31 (0.14-0.69)	0.004	0.30 (0.15-0.60)	0.001	0.30 (0.15-0.57)	<0.001
**12 months**	0.41 (0.22-0.75)	0.004	0.35 (0.20-0.60)	<0.001	0.34 (0.19-0.58)	<0.001	0.49 (0.23-1.04)	0.06	0.40 (0.21-0.77)	0.006	0.37 (0.20-0.70)	0.002

[a] Backwards elimination models including age, sex, initial GCS-score, injury severity score, coagulation factors, thrombocytopenia, and initial hemorrhage volume

© 2021 Jha RM et al. *JAMA Network Open*.

eTable 6. Genotype Frequencies of *ABCC8* and *TRPM4* SNPs Associated With Hemorrhage Progression in TBI

SNP Details	Sample	Homozygous Wild-type n (%)	Heterozygous n (%)	Homozygous Variant n (%)	p-value (Fisher)
ABCC8					
rs2237982 Intron 10 Wild-type: C Variant: T MAF: 0.424	Full Cohort	106 (32.7%)	161 (49.7%)	57 (17.6%)	-
	No-Craniectomy subcohort	73 (31.2%)	120 (51.3%)	41 (17.5%)	-
	- Hemorrhage Progression-Yes	30/73 (41.1%)	45/120 (37.5%)	27/41 (65.9%)	0.006
rs2283261 Intron 10 Wild-type: A Variant: C MAF: 0.404	Full Cohort	97 (33.0%)	157 (53.4%)	40 (13.6%)	-
	No-Craniectomy subcohort	64 (30.8%)	117 (56.3%)	27 (13.0%)	-
	- Hemorrhage Progression-Yes	26/64 (40.6%)	46/117 (39.3%)	19/27 (70.4%)	0.012
rs3819521 Intron 3 Wild-type: C Variant: T MAF: 0.340	Full Cohort	123 (41.8%)	142 (48.3%)	29 (9.9%)	-
	No-Craniectomy subcohort	82 (39.4%)	105 (50.5%)	21 (10.1%)	-
	- Hemorrhage Progression-Yes	35/82 (42.7%)	42/105 (40.0%)	14/21 (66.7%)	0.084
rs8192695 Exon 3 Wild-type: G Variant: A MAF: 0.049	Full Cohort	266 (90.5%)	27 (9.2%)	1 (0.3%)	-
	No-Craniectomy subcohort	170 (90.4%)	17 (9.0%)	1 (0.5%)	-
	- Hemorrhage Progression-Yes	69/170 (40.6%)	11/17 (64.7%)	1/1 (100%)	.052
TRPM4					
rs3760666 Intron 2 Wild-type: T Variant: C MAF: 0.335	Full Cohort	126 (42.9%)	139 (47.3%)	29 (9.9%)	-
	No-Craniectomy subcohort	88 (46.8%)	88 (46.8%)	12 (6.4%)	-
	- Hemorrhage Progression-Yes	46/88 (52.3%)	31/88 (35.2%)	4/12 (33.3%)	0.060
rs1477363 Intron 6 Wild-type: C Variant: A MAF: 0.276	Full Cohort	151 (51.4%)	124 (42.2%)	19 (6.5%)	-
	No-Craniectomy subcohort	104 (55.3%)	74 (39.4%)	10 (5.3%)	-
	- Hemorrhage Progression-Yes	53/104 (51.0%)	24/74 (32.4%)	4/10 (40%)	0.042
rs10410857 Intron 9 Wild-type: G Variant: A MAF: 0.333	Full Cohort	129 (43.9%)	134 (45.6%)	31 (10.5%)	-
	No-Craniectomy subcohort	90 (47.9%)	84 (44.7%)	14 (7.5%)	-
	- Hemorrhage Progression-Yes	50/90 (55.6%)	27/84 (32.1%)	4/14 (28.6%)	0.004
rs909010 Intron 12 Wild-type: T Variant: C MAF: 0.357	Full Cohort	119 (40.5%)	140 (47.6%)	35 (11.9%)	-
	No-Craniectomy subcohort	81 (43.1%)	87 (46.3%)	20 (10.6%)	-
	- Hemorrhage Progression-Yes	48/81 (59.3%)	25/87 (28.7%)	8/20 (40%)	<0.001

© 2021 Jha RM et al. *JAMA Network Open*.

eTable 7. *ABCC8* and *TRPM4* Single-Nucleotide Polymorphisms Associated With Quantitative Change in Intraparenchymal Hemorrhage Volumes in Severe TBI							
**SNP**	**Model**	**Hemorrhage Progression β (95% CI, p-value)**					
		**6-hour**	***p-value***	**24-hour**	***p-value***	**5-day**	***p-value***
ABCC8							
	Additive (Reference GG)						
rs8192695[b,c]	GA	0.19 (-0.29 – 0.67)	0.44	0.75 (0.25 – 1.24)	0.003[a]	0.57 (0.08 – 1.06)	0.02
	AA	1.82 (0.22-3.20)	0.02	1.49 (-0.28-3.27)	0.10	1.43 (-0.33-3.20)	0.11
	Dominant GA, AA	0.34 (-0.13 – 0.81)	0.15	0.80 (0.32 – 1.28)	0.001[a]	0.63 (0.15 – 1.11)	0.01
rs1799859	Additive (Reference CC)						
	CT	0.02 (-0.23 – 0.27)	0.87	0.34 (0.04 – 0.60)	0.03	0.33 (0.04 – 0.62)	0.02
	TT	0.12 (-0.60 – 0.83)	0.75	0.74 (0.15 – 1.34)	0.02	1.03 (0.31 – 1.77)	0.006[a]
	Dominant CT, TT	0.03 (-0.22 – 0.27)	0.82	0.39 (0.11 – 0.67)	0.007[a]	0.40 (0.12 – 0.69)	0.006[a]
rs4148640[a]	Additive (Reference GG)						
	GT	0.04 (-0.21 – 0.30)	0.73	0.34 (0.04 – 0.63)	0.03	0.35 (0.06 – 0.64)	0.02
	TT	0.12 (-0.59 – 0.84)	0.73	0.74 (0.15 – 1.34)	0.02	1.05 (0.31 – 1.78)	0.005[a]
	Dominant GT, TT	0.05 (-0.20 – 0.30)	0.69	0.39 (0.11 – 0.67)	0.007[a]	0.42 (0.14 – 0.70)	0.004[a]
TRPM4							
rs3760666[b]	Additive (Reference TT)						
	TC	-0.28 (-0.53 --0.03)	0.03	-0.18 (-0.47 – 0.12)	0.24	-0.18 (-0.48 – 0.12)	0.24
	CC	-0.40 (-0.92 – 0.12)	0.14	-0.49 (-1.16 – 0.19)	0.16	-0.29 (-0.92 – 0.35)	0.37
	Dominant TC,CC	-0.29 (-0.54 --0.05)	0.02	-0.21 (-0.50 – 0.08)	0.16	-0.19 (-0.48 – 0.10)	0.19
rs1477363[b]	Additive (Reference CC)						
	CA	-0.26 (-0.52 - -0.01)	0.05	-0.24 (-0.53 – 0.06)	0.11	-0.27 (-0.57 – 0.02)	0.07
	AA	-0.31 (-0.89 – 0.26)	0.28	-0.37 (-1.21 – 0.46)	0.38	-0.28 (-0.94 – 0.37)	0.39
	Dominant CA, AA	-0.27 (-0.52 - -0.02)	0.03	-0.25 (-0.53 – 0.04)	0.09	-0.28 (-0.56 – 0.01)	0.06
rs10410857[b,c]	Additive (Reference GG)						
	GA	-0.29 (-0.55 - -0.04)	0.02	-0.35 (-0.64 - -0.06)	0.02	-0.42 (-0.71 - -0.13)	0.005[a]
	AA	-0.44 (-0.91 - .03)	0.07	0.61 (-1.24 – 0.02)	0.06	-0.50 (-1.09, 0.08)	0.09
	Dominant GA, AA	-0.32 (-0.56 - -0.08)	0.01	-0.38 (-0.66 - -0.09)	0.009[a]	-0.43 (-0.71 - -0.15)	0.003[a]
rs909010[b,c]	Additive (Reference TT)						
	TC	-0.41 (-0.66 - -0.16)	0.002[a]	-0.42 (-0.71 - -0.12)	0.006[a]	-0.49 (-0.78 - -0.19)	0.002[a]
	CC	-0.37 (-0.79 – 0.05)	0.09	-0.47 (-1.00 – 0.06)	0.08	-0.47 (-0.97 – 0.03)	0.07
	Dominant TC, CC	-0.40 (-0.64 - -0.16)	0.001[a]	-0.43 (-0.71 - -0.14)	0.003[a]	-0.48 (-0.77 - -0.20)	0.001[a]
rs8104571[d]	Additive (Reference CC)						
	CT	-0.26 (-1.01 – 0.50)	0.51	1.15 (-.23 – 2.06)	0.01	1.09 (-.20 – 1.99)	0.02
	TT	-		-		-	

[a]p< 0.00931 meeting significance after Benjamin-Yekutieli correction for multiple comparisons
[b] SNPs are significant predictors of hemorrhage expansion (binary, Table 1).
[c]SNPs are significant (p<0.05) in all-comers regardless of craniectomy status (Supplemental Table 9)
[d] SNPs previously reported to be predictive of intracranial pressure and/or acute CT edema after TBI.

© 2021 Jha RM et al. *JAMA Network Open*.

**eTable 8.** ***ABCC8*** **and** ***TRPM4*** **Single-Nucleotide Polymorphisms Associated With Intraparenchymal Hemorrhage Progression in Severe TBI (Full Cohort)**							
**SNP**	**Model**	**Intraparenchymal Hemorrhage Progression OR (95% CI)**					
		**6-hour**	***p-value***	**24-hour**	***p-value***	**5-day**	***p-value***
ABCC8							
	Additive (Reference GG)						
rs8192695[b,c]	GA<br>AA	2.98 (1.11-8.03)<br>-	0.03	2.38 (0.97-5.84)<br>-	0.06	2.56 (1.01-6.49)<br>-	0.05
	Dominant<br>GA, AA	3.29 (1.24-8.71)	0.02	2.63 (1.09-6.38)	0.03	2.82 (1.12-7.10)	0.03
rs1799857[d]	Additive (Reference CC)						
	CT<br>TT	1.12 (0.61-2.37)<br>3.14 (1.34-7.38)	0.60<br>0.009[a]	0.90 (0.50-1.62)<br>2.23 (1.05-4.76)	0.73<br>0.04	0.81 (0.45-1.46)<br>1.85 (0.86-3.99)	0.48<br>0.11
	Recessive<br>TT	2.79 (1.33-5.85)	0.007[a]	2.38 (1.22-4.64)	0.01	2.10 (1.07-4.15)	0.03
TRPM4							
	Additive (Reference GG)						
rs10410857[b,c]	GA<br>AA	0.50 (0.27-0.94)<br>0.71 (0.26-1.95)	0.03<br>0.50	0.44 (0.26-0.76)<br>0.48 (0.18-1.24)	0.003[a]<br>0.13	0.42 (0.25-0.73)<br>0.42 (0.16-1.09)	0.002[a]<br>0.07
	Dominant<br>GA, AA	0.54 (0.30-0.97)	0.04	0.45 (0.26-0.76)	0.003[a]	0.42 (0.25-0.71)	0.001[a]
	Additive (Reference TT)						
rs909010[b,c]	TC<br>CC	0.48 (0.26-0.90)<br>0.75 (0.28-1.97)	0.02<br>0.56	0.42 (0.24-0.73)<br>0.54 (0.22-1.33)	0.002[a]<br>0.18	0.38 (0.22-0.66)<br>0.50 (0.22-1.23)	0.001[a]<br>0.13
	Dominant<br>TC, CC	0.52 (0.29-0.95)	0.04	0.44 (0.26-0.75)	0.002[a]	0.40 (0.24-0.67)	0.001[a]

[a]p< 0.00931 meeting significance after Benjamin-Yekutieli correction for multiple comparisons,
[b]SNPs that are significant predictors of hemorrhage expansion (binary, Table 1).
[c]SNPs that are significant (p<0.05) predictors of quantitative contusion expansion volumes (continuous variable, Supplemental Table 7)
[d]SNPs previously reported to be predictive of intracranial pressure and/or acute CT edema after TBI.

© 2021 Jha RM et al. *JAMA Network Open*.

eTable 9. ***ABCC8* and *TRPM4* Haplotypes Associated With Contusion Expansion in Severe TBI**							
**Haplotype**	**Model**	**Hemorrhage Progression OR (95% CI, p-value)**					
		**6-hour**	***p-value***	**24-hour**	***p-value***	**5-day**	***p-value***
ABCC8 rs2237982 (C/T) - rs2283261 (A/C) - rs8192695 (G/A)- rs3819521 (C/T)							
TCAC	Additive	3.78 (1.25, 11.48)	0.02	3.37 (1.24-9.14)	0.02	2.80 (1.04-7.59)	0.04
	Dominant	3.02 (1.00-9.11)	0.05	2.72 (1.02-7.29)	0.05	2.24 (0.94-6.00)	0.11
TCA-	Additive	3.88 (1.27-11.86)	0.02	3.34 (1.23-9.04)	0.02	2.78 (1.03-7.53)	0.04
-CA-	Additive	5.25 (1.72-16.04)	0.004[a]	3.87 (1.42-10.53)	0.008[a]	3.25 (1.19-8.84)	0.02
TRPM4 rs3760666 (T/C) – rs1477363 (C/A) – rs10410857 (G/A) – rs909010 (T/C)							
CAAC	Additive	0.58 (0.32-1.03)	0.06	0.52 (0.31-0.86)	0.01	0.54 (0.33-0.89)	0.02
	Dominant	0.46 (0.24-0.94)	0.03	0.46 (0.26-0.84)	0.01	0.48 (0.27-0.84)	0.01
-AAC	Additive	0.57 (0.32-1.02)	0.06	0.51 (0.30-0.84)	0.009[a]	0.53 (0.33-0.86)	0.01
	Dominant	0.47 (0.24-0.93)	0.03	0.45 (0.25-0.82)	0.008[a]	0.46 (0.26-0.81)	0.007[a]
C-AC	Additive	0.57 (0.33-0.99)	0.04	0.49 (0.30-0.79)	0.004[a]	0.52 (0.33-0.82)	0.005[a]
	Dominant	0.50 (0.26-0.96)	0.04	0.46 (0.26-0.80)	0.006[a]	0.48 (0.28-0.83)	0.008[a]
- - AC	Additive	0.54 (0.31-0.92)	0.02	0.46 (0.28-0.73)	0.001[a]	0.48 (0.30-0.75)	0.001a
	Dominant	0.48 (0.25-0.91)	0.02	0.43 (0.25-0.75)	0.003[a]	0.45 (0.26-0.77)	0.003a
ABCC8 rs2283261 (A/C) - rs8192695 (G/A) and TRPM4 rs10410857 (G/A) – rs909010 (T/C)							
AGAC	Additive	0.43 (0.18-1.0)	0.05	0.28 (0.13-0.62)	0.002[a]	0.28 (0.13-0.60)	0.001[a]
CAGT	Additive	3.73 (1.24-11)	0.02	2.67 (0.99-7.26)	0.05	2.17 (0.8-5.89)	0.12

CI= confidence interval; NS= not significant; OR= odds ratio

[a]p< 0.00931 meeting significance after Benjamin-Yekutieli correction for multiple comparisons

© 2021 Jha RM et al. *JAMA Network Open*.

**eTable 10. Haplotype Distribution in the Cohort of Severe TBI**<br><br>*SNP order (0=wild type, 1= variant):*<br>*ABCC8: rs2237982-rs8192695-rs2283261-rs3819521-*<br>*TRPM4: rs909010-rs10410857-rs1477363-rs3760666*	
**Haplotype**	**Frequency (proportion)**
00000000	0.364198
00000001	0.012795
00001000	0.024099
00001100	0.0184
00001111	0.177303
00010000	0.008384
10100000	0.012072
10101111	0.010521
10110000	0.195595
10111000	0.009866
10111100	0.010973
10111101	0.033903
10111111	0.079035
11100000	0.017407
11101111	0.009391

© 2021 Jha RM et al. *JAMA Network Open*.

**eTable 11. Intraparenchymal Hemorrhage Progression Risk Polymorphisms Associated With 6-mo Glasgow Outcome Scale (GOS) Score**				
**SNP and model**		**Common OR[a,b] (95% CI)**	***p-value (model)***	***p-value (LR-test vs clinical only)***
ABCC8				
rs2237982	Ref (GG or GA)			
Recessive	AA	0.45 (0.23-0.91)	0.03[b]	0.02[b]
rs2283261	Ref (CC or CT)			
Recessive	TT	0.45 (0.22-0.90)	0.02[b]	0.02[b]
rs8192695	Ref (CC)			
Dominant	CT or TT	0.38 (0.02-9.29)	0.55	0.55
rs3819521	Ref (GG or GT)			
Recessive	TT	0.44 (0.20-0.97	0.04[b]	0.04[b]

LR-test: likelihood ratio test of model including SNP vs model with only clinical covariates
[a]Odds of moving one point higher on the GOS scale
[b]Statistically significant at *p<0.05*

© 2021 Jha RM et al. *JAMA Network Open*.

eTable 12. Sample Size Calculations for Genotype-Based Patient Selection in Clinical Trial

**Group**	**Probability of Progression**	**Percent of All Comers**	**Sample size Required ***	**Number Screened**
All comers	43.3%	100%	576	576
At least one *ABCC8* variant (risk) SNP	62.5%	26.9%	296	1100
At least one *TRPM4* wild-type (risk) SNP	52.9%	58.2%	408	701
At least one *ABCC8* variant (risk) SNP AND at least one *TRPM4* wild-type (risk) SNP	83.3%	11.5%	136	1183
At least one *ABCC8* variant (risk) SNP OR at least one *TRPM4* wild-type (risk) SNP	51.7%	73.6%	428	582
Clinical Model Alone[#]	72.2%	17.3%	208	1202
Clinical Model + Genotypes[#]	61.9%	50.4%	300	594

*Sample size for relative risk reduction of 30% in hemorrhage progression, power of 0.9

[#] Subgroup of patients where the model predicts >50% risk of progression. Clinical models contain the covariates from the backward elimination multivariable regression models (age, age, sex, initial GCS-score, injury severity score, coagulation factors, thrombocytopenia, and initial hemorrhage volume).

© 2021 Jha RM et al. *JAMA Network Open*.

eTable 13. Regulatory Annotations of *ABCC8* and *TRPM4* Single-Nucleotide Polymorphisms Associated With Hemorrhage Progression in Severe TBI

(Includes data for high-probability causal SNPs for proxy-SNPs in LD, r^2>0.8)

Genotyped SNP	High Probability Causal SNP in LD (r^2)	Regu-lomeDB Score	Promoter Histone Marks	Enhancer Histone Marks	DNAse	Proteins Bound[b] (ChIP data)	Transcription Factors with Regulatory Motifs Altered[c] (HaploReg PWM score)
***ABCC8* SNPs**							
rs8192695		0.135	N	N	N	-	HIC1(-2.8), LBP1 (1.6), NANOG (9.4), TCF12 (-2.6), ZFP410 (6.9)
	rs77462644 (1.0)	0.609	Y	Y	Y	ATF7, CTBP1, DPF2, EGR2, FOXJ2, GATA2, HDAC1, MAX, MAZ, MYC, PATZ, POL2, POLR2A, TCF7L2, USF2, VEZF1, ZFN629	-
rs3819521[a]		0.135	Y	Y	Y		FOXA (11.9), NF-κB (-3.6) , STAT (12.0)
	rs4148610 [a] (0.96)	0.719	Y	Y	Y	ATF7, CTBP1, DPF2, EGR2, EMSY, EP300, GATA2, HDAC, JUND, MAX, MAZ, MYC, PATZ1, POLR2A, PRDM1, PRDM10, SP7, TCFL2, USF2, VEZF1, ZNF316, ZNF398, ZNF629	HOXA13 (10.6), PAX3 (3.2), PAX5 (1.6)
	rs4148609 [a] (0.92)	0.144	Y	Y	Y	ATF7, CTBP1, DPF2, GR2, EMSY, EP300, FOXJ2, GATA2, HDAC1, JUND, KLF1, MAX, MAZ, MYC, PATZ1, POL2, POLR2A, PRDM1, PRDM10, SP7, TCF7L2, USF2, VEZF1, ZFN316, ZFN629	ETS1
	rs2301703 [a,e] (0.81)	0.609	Y	Y	Y	ATF1, CTBP1, FOXJ2, GATA2, HDAC1, KDM1A, MAX, POL2, POL2RA, VEZF1	CACD (-2.6), MYC (-1.8), MYF (-4.0), NRSF (-3.2), SMC3 (-3.9), SIN3Ak (-8.2), TCF12 (11.3)
	rs2283254 [a] (0.95)	0.609	Y	Y	Y	ARID3A, ATF3, CTBP1, CTCF, EMSY, MAFK, MAZ, MYC, NFYA, NR3C1, POLR2A, RAD21, REST, SMARCAS, SMC3, TEAD4, TRIM22, XRCC5, YY1, ZFN143, ZFN316, ZFN444, ZFN600	EVI (12.0)
	rs3815066 (0.96)	0.609	Y	Y	Y	ATF7, CTBP1, DPF2, EGR2, EMSY, EP300, FOXJ2, GATA2, HDAC1, JUND, KLF1, MAX, MAZ, MYC, PATZ1, POL2, POLR2A, PRDM10, SP7, TCFL2, USF2, VEZF1, ZNF316, ZNF629	EBF (-0.8), ER-α (–0.6), PLAG1 (-10.8)
rs2237982[#*] and rs2283261[#*]		0.599<br>0.609	Y<br>Y	Y<br>Y	Y<br>Y	-<br>GR, FOS, NR3C1, RFX3	MEF2D, MYEF2, MYEF2-B<br>-
	rs2237980 (1.0)	0.568	Y	Y	Y	94 reports of proteins bound including FOS, PPAR-γ, SP1 multiple zinc fingers.	ARNT2 (-12), BHLHE40 (-11.9), E2A (11.9), MXI1 (-0.1), MYC (-11.4), SIN3Ak (-4.2), ZEB1 (12)
	rs2301703 [a,d] (0.92)	0.609	Y	Y	Y	see entry for rs2301703 above	see entry for rs2301703 above
***TRPM4* SNPs**							
rs3760666	-	0.609	N	Y	Y	ZNF512, ZNF394, ZNF596	-
rs1477363	-	0.5896	N	Y	N	SPI1, ZNF121	NR2F2 (-11.7), TATA (11.7)
rs10410857	-	0.329	N	N	N	-	ARHGEF12, BCL6β (–1.7)
rs909010		0.609	N	Y	N	NR2F2	GR (-0.6), HNF4 (2.9), IRF (-1.6), RXRA (-0.1)

© 2021 Jha RM et al. *JAMA Network Open*.

	rs34639121 (0.82)	0.83	Y	Y	Y	>250 reports of proteins bound including ATF1, CTCF, MYC, PPAR-γ	P53 (-12)

LD= linkage disequilibrium; PWM= position weight matrix log odds scores from HaploReg V4.1; SNP= single nucleotide polymorphism

[a] SNPs are previously reported as significant predictors of post traumatic ICP and/or acute CT edema.

[b] Obtained from RegulomeDB 2.0 and HaploReg V4.1.

[c] Obtained from RegulomeDB 2.0 and HaploReg V4.1. PWM log-odds score obtained from HaploReg V4.1 annotating SNP effects on regulatory motifs and transcription factor binding affinity: Log Odds Variant– Log Odds Reference allele. An increase in log-odds scores suggest increased transcription factor binding strength based on PWMs collected by HaploReg from TRANSFAC, JASPAR and Protein Binding Microarray experimental data. Absolute values for log-odds scores for the reference and variant alleles are also available from HaploReg.

[d]Proxy SNP in LD with more than 1 genotyped SNP

© 2021 Jha RM et al. *JAMA Network Open*.

eTable 14. Association of *ABCC8* and *TRPM4* Single-Nucleotide Polymorphisms With IPH Progression (Quantitative Definition Only)							
**SNP**	**Model**	**Hemorrhage Progression OR (95% CI)**					
		**6-hour IPH Progression**	***p-value***	**24-hour IPH Progression**	***p-value***	**120-hour IPH Progression**	***p-value***
ABCC8							
	Additive (Reference CC)						
rs2237982	CT<br>TT	0.91 (0.46-1.78)<br>2.07 (0.91-4.73)	0.78<br>0.08	0.97 (0.53-1.76)<br>2.61 (1.21-5.64)	0.91<br>0.02	0.89 (0.49-1.59)<br>2.08 (0.97-4.46)	0.68<br>0.06
	Variant recessive<br>TT	2.20 (1.07-4.52)	0.03	2.67 (1.36-5.23)	0.004	2.24 (1.15-4.39)	0.02
rs2283261	Additive (Reference AA)						
	AC<br>CC	1.30 (0.62-2.73)<br>2.81 (1.00-7.89)	0.48<br>0.05	1.20 (0.63-2.27)<br>2.85 (1.16-7.02)	0.58<br>0.02	1.24 (0.66-2.33)<br>2.55 (1.04-6.25)	0.96<br>0.04
	Variant recessive<br>CC	2.36 (0.95-5.87)	0.06	2.66 (1.19-5.95)	0.02	2.22 (1.00-4.94)	0.05
rs8192695	Additive (Reference GG)						
	GA<br>AA	2.71 (0.92-8.00<br>-	0.07	2.25 (0.83-6.16)<br>-	0.11	1.98 (0.72-5.38)<br>-	0.18
	Variant dominant<br>GT or TT	3.24 (1.12-9.33)	0.03	2.55 (0.95-6.85)	0.06	2.23 (0.83-5.99)	0.11
rs3819521	Additive (Reference CC)<br>CC	0.80 (0.40-1.59)	0.52	0.87 (0.48-1.58)	0.64	0.97 (0.54-1.76)	0.92
	CT	1.67 (0.56-4.95)	0.36	2.46 (0.95-6.36)	0.06	2.15 (0.83-5.54)	0.11
	Variant recessive<br>TT	1.87 (0.67-5.26)	0.23	2.65 (1.08-6.51)	0.03	2.18 (0.89-5.36)	0.09
TRPM4							
rs3760666	Additive (Reference TT)						
	TC<br>CC	0.72 (0.37-1.43)<br>0.54 (0.12-2.41)	0.35<br>0.42	0.61 (0.34-1.10)<br>0.34 (0.09-1.25)	0.10<br>0.10	0.66 (0.37-1.18)<br>0.32 (0.09-1.17)	0.16<br>0.14
	Variant dominant<br>TC or CC	0.70 (0.36-1.35)	0.29	0.57 (0.33-1.01)	0.06	0.61 (0.35-1.07)	0.09
rs1477363	Additive (Reference CC)						
	CA<br>AA	0.51 (0.31-1.22)<br>0.59 (0.11-3.05)	0.16<br>0.53	0.51 (0.28-0.93)<br>0.45 (0.11-1.84)	0.03<br>0.27	0.57 (0.32-1.03)<br>0.42 (0.10-1.71)	0.06<br>0.23
	Variant dominant<br>CA or AA	0.51 (0.31-1.20)	0.15	0.51 (0.28-0.90)	0.02	0.56 (0.32-0.98)	0.04
rs10410857	Additive (Reference GG)						
	GA<br>AA	0.67 (0.34-1.32)<br>0.41 (0.09-1.84)	0.25<br>0.25	0.46 (0.25-0.82)<br>0.26 (0.07-0.94)	0.009<br>0.04	0.50 (0.28-0.89)<br>0.24 (0.07-0.87)	0.02<br>0.03
	Variant dominant<br>GA or AA	0.63 (0.33-1.23)	0.18	0.43 (0.24-0.75)	0.003	0.46 (0.26-0.81)	0.007
rs909010	Additive (Reference TT)						
	TC<br>CC	0.59 (0.30-1.18)<br>0.75 (0.22-2.54)	0.14<br>0.65	0.51 (0.28-0.93)<br>0.49 (0.17-1.39)	0.03<br>0.18	0.55 (0.31-0.99)<br>0.46 (0.16-1.31)	0.05<br>0.15
	Variant dominant<br>TC or CC	0.62 (0.32-1.19)	0.15	0.51 (0.29-0.90)	0.02	0.54 (0.31-0.94)	0.03

© 2021 Jha RM et al. *JAMA Network Open*.

eFigure 1. Opposite Associations of *ABCC8* vs *TRPM4* variant SNPs with Intraparenchymal Hemorrhage Progression in TBI

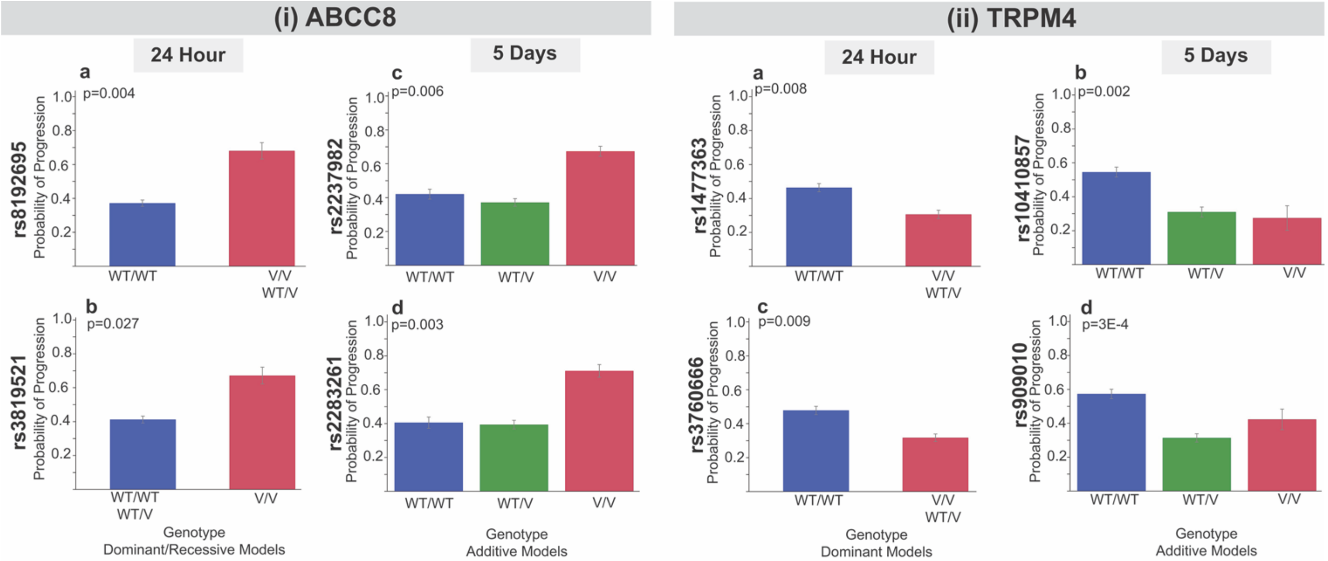

Panel of bar charts graphically depicting examples of the opposite directions of associations of *ABCC8* (A and B) vs *TRPM4* (C and D) variant SNPs with odds of hemorrhage progression in TBI at 24 hours and 5 days. Probability of progression is on the y-axis, and genotypes are on the x-axis. A dominant model for *ABCC8* SNP rs8192695 (A-i) shows increased probability of 24h hemorrhage progression with presence of the variant SNP (red) vs homozygous wild-type (blue, *P* = .004). A recessive model for rs3812695 (A-ii) shows increased probability of 24-hour progression in homozygous variants (red, *P* = .027). Additive models for rs2237982 (*P* = .006) and rs2283261 (*P* = .003) show increased probability of 5-day hemorrhage progression in homozygous variants (red) compared with both heterozygotes (green) and homozygous wild-type (blue). Conversely, dominant models at 24h for *TRPM4* SNPs rs1477363 (C-i, *P* = .008) and rs3760666 (C-ii, *P* = .009) show decreased probability of hemorrhage progression with presence of the variant SNP (red) vs homozygous wild-type (blue) at 24h. Additive models for rs10410857 (D-i, *P* = .002) and rs909010 (D-ii, *P* = 3E-4) demonstrate decreased probability of hemorrhage progression in homozygous variants (red) vs both heterozygotes (green) and homozygous wild-type (blue).

© 2021 Jha RM et al. *JAMA Network Open*.

eFigure 2. Spatial Distribution of *TRPM4* SNPs in Hemorrhage Progression After TBI

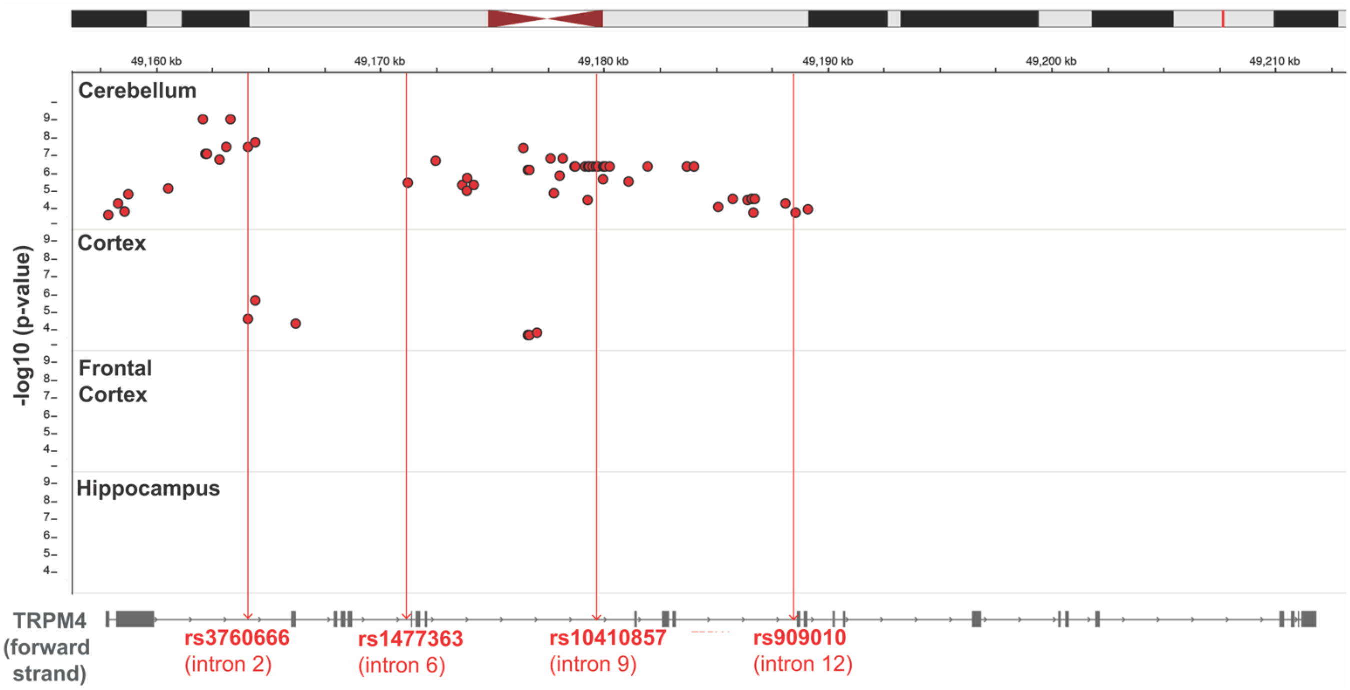

Chromosomal Location and Identification of *TRPM4* SNPs as Brain eQTLs. This graph shows the -log10 (*P* value) of significant cis-expression quantitative trait loci (eQTLs, red dots) within the *TRPM4* gene (y-axis) and their location on the gene (x-axis). Subgraphs demonstrate the single nucleotide polymorphism eQTL *P* values and chromosomal locations based on different tissue isolates from the genotype-tissue expression (GTEx) project with brain-specific eQTLs in the cerebellum, cortex, frontal cortex, and hippocampus. No trans-eQTLs are present. *TRPM4* is encoded on the forward strand with exons delineated by vertical gray bars; exon 1 is therefore leftmost and exon 25 is located on the far right of the x-axis. Evident from the graph, majority of brain-specific cis-eQTLs are located upstream of exon 12 including the four significant *TRPM4* SNPs associated with hemorrhage progression, rs3760666, rs1477363, rs10410857, rs909010 – these are highlighted by the vertical red lines.

© 2021 Jha RM et al. *JAMA Network Open*.

eFigure 3. Variant *TRPM4* SNPs Associated With Decreased Hemorrhage Progression in TBI are eQTL With Decreased *TRPM4* Expression in Cerebellum

© 2021 Jha RM et al. *JAMA Network Open*.

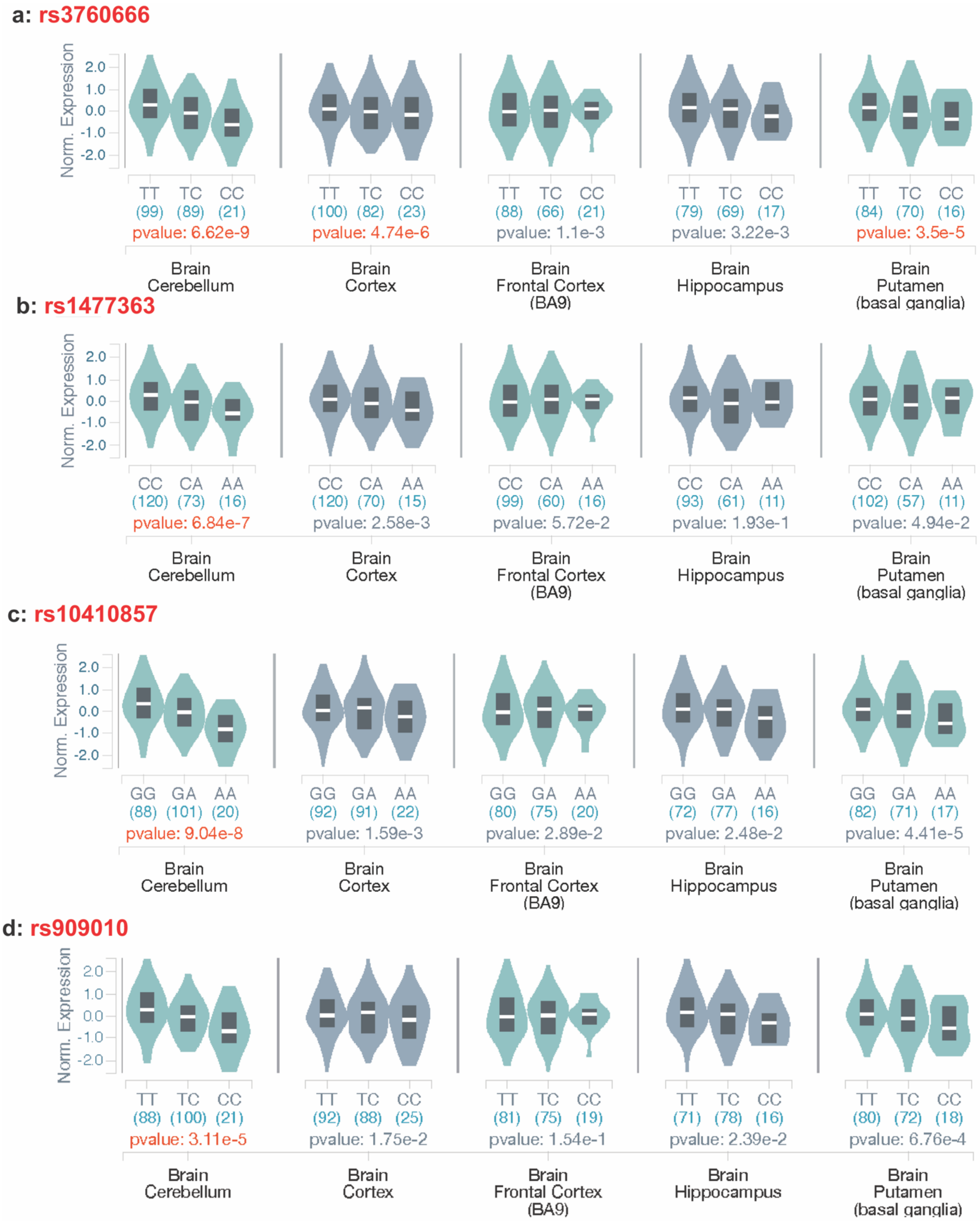

© 2021 Jha RM et al. *JAMA Network Open*.

Panel demonstrating violin plots of normalized mRNA expression levels (y-axis) associated with the genotypes (x-axis) of four *TRPM4* SNPs significantly associated with hemorrhage progression after severe traumatic brain injury (TBI). Each subpanel shows the normalized *TRPM4* mRNA expression level in the cerebellum, cortex, frontal cortex, hippocampus and putamen by SNP genotype: rs3760666 (A), rs1477363 (B), rs10410857 (C) and rs909010 (D). Shaded regions in teal or blue (alternating between brain region) of the individual violin plots indicate the density distribution of mRNA expression in the samples in each respective genotype, with the white line showing the median value. The *P* value provided for each SNP at each location indicates the *P* value for different expression levels across genotypes for that SNP in the respective tissue location. Unlike *ABCC8*, all four *TRPM4* SNPs are brain-specific eQTLs only in the cerebellum, with rs3760666 also having significantly different *TRPM4* mRNA expression levels in the brain cortex and putamen. In all cases, mRNA expression is lower with variant *TRPM4* SNPs, with a dose-dependent effect noted between homozygous wild-type, heterozygotes, and homozygous variants.

© 2021 Jha RM et al. *JAMA Network Open*.

eFigure 4. Sequence Logos Demonstrating Impact of *ABCC8* and *TRPM4* SNPs on Transcription Factor Motifs

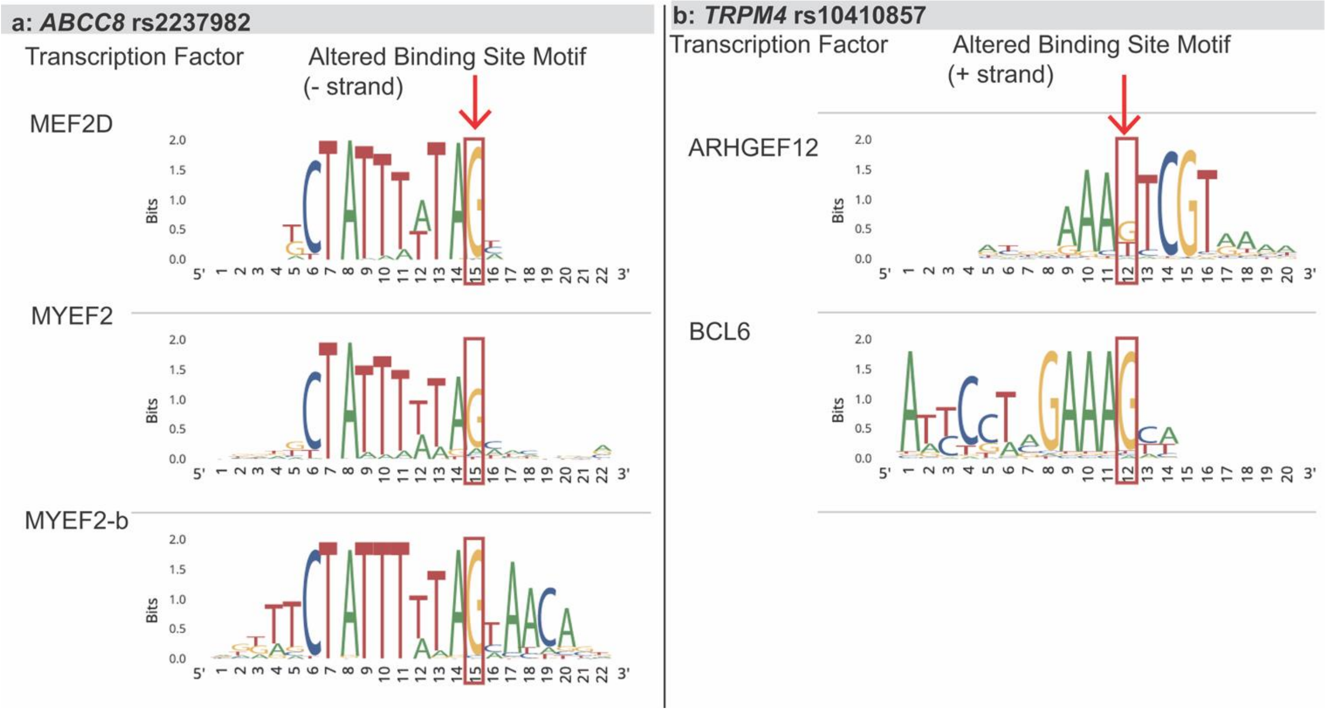

Schematics of sequence logos of different transcription factor binding site motifs obtained from RegulomeDB annotations for *ABCC8* SNP rs2237982 (A) and *TRPM4* SNP rs10410857. The y-axis in each subgraph represents a binary information tool (bits) containing two pieces of information: the height of the base pair alphabet is non linearly proportional to the frequency with which it is found at that position, and the total height of each column denotes the importance/strength of that location for transcription factor recognition and binding. Each base pair is shown in a specific color. The location altered by the SNP is highlighted in a red box. For transcription factors MEF2d, MYEF2, and MYEF2-b, *ABCC8* SNP rs2237982 changes a highly conserved base-pair, and this site is highly important for determining strength of transcription factor recognition and binding. *TRPM4* SNP rs10410857, only moderately affects transcription factor binding for ARHGEF12 but strongly affects BCL6.

© 2021 Jha RM et al. *JAMA Network Open*.

eFigure 5. Brain Chromatin States of *ABCC8* and *TRPM4* Genomic Loci

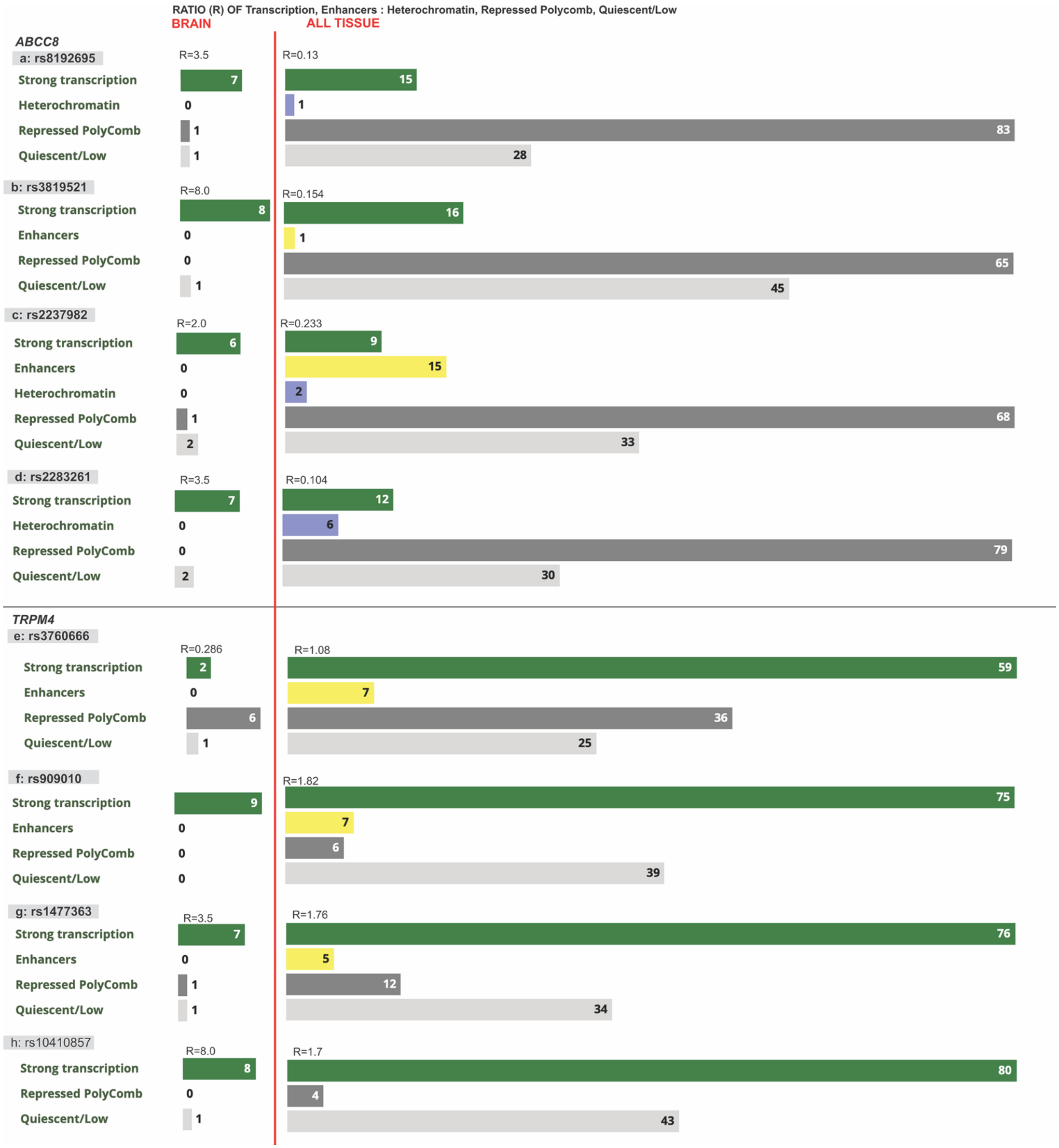

Annotations from RegulomeDB showing chromatin states in regions of DNA containing significant *ABCC8* and *TRPM4* SNPs (200 bp bin region in the genome). The vertical red line separates chromatin states in brain tissue (left) from all-tissue samples (right). The horizontal black line separates *ABCC8* SNPs (A: rs8192695, B: rs3819521, C: rs2237982, D: rs2283261) from *TRPM4* SNPs (E: rs3760666, F: rs909010, G: rs1477363, H: rs10410857). The number of samples with strong transcription (green),

enhancers (yellow), heterochromatin (lavendar), repressed polycomb (dark gray) and quiescent/low transcription (light gray) is shown for each SNP in brain vs all-tissue. For each SNP, the ratio (R) of strong transcription and enhancers to repressed polycomb, quiescent states, and heterochromatin is shown. For example, for all *ABCC8* SNPs, R is markedly higher in brain tissue vs other tissue, with most regional samples demonstrating strong transcriptional activity. This difference was not as pronounced with TRPM4 which was annotated to have strong transcription (green) in all tissues.

© 2021 Jha RM et al. *JAMA Network Open*.

eFigure 6. Linkage Disequilibrium Maps for *ABCC8* and *TRPM4* SNPs Regional Loci

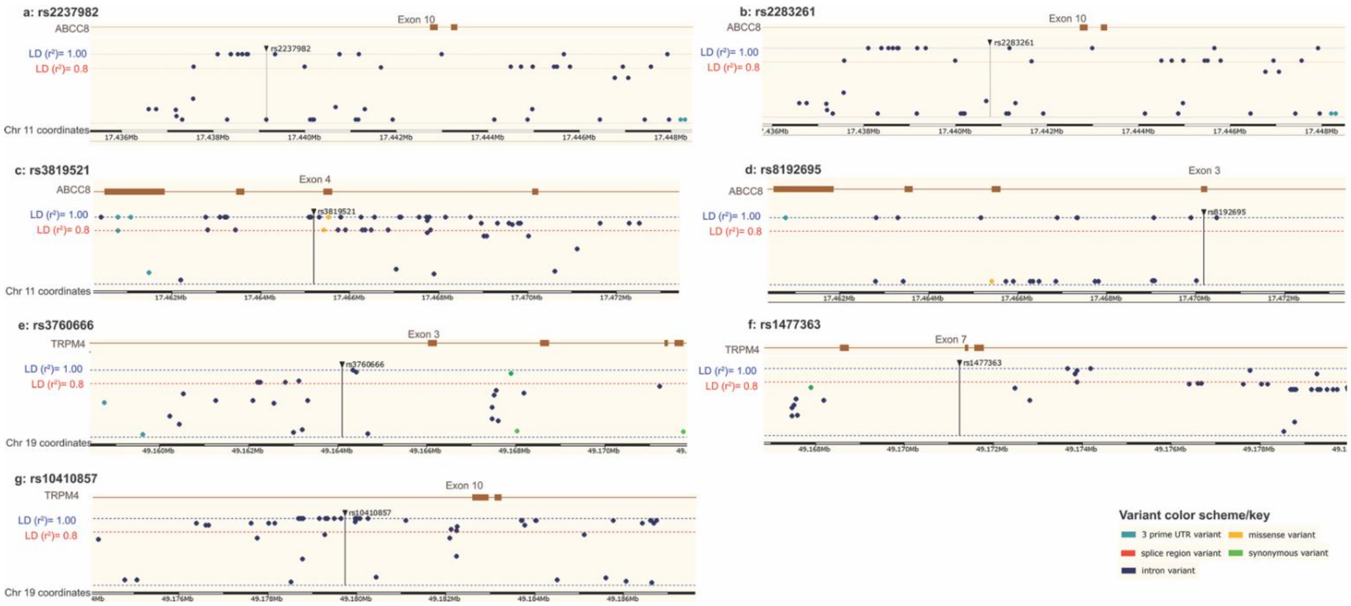

Linkage disequilibrium (LD) plots from the Ensembl genome browser of significant *ABCC8* (A: rs2237982, B: rs2283261, C: rs3819521, D: rs8192695) and TRPM4 (E: rs3760666, F: rs1477363, and G: rs10410857) SNPs. Zoomed in chromosomal coordinates are shown for each SNP at the base of each image (x-axis), with corresponding locations of exons marked by an orange box on the top of each image. The y-axis is the r^2 value quantifying the extent (correlation coefficient) of LD. The threshold for perfect LD (r^2 = 1.0) is marked by a blue dotted horizontal line and LD (r^2 = 0.8) is marked by a red dotted horizontal line on each subplot. Proxy SNPs based on location and LD are shown by dots. The variant key is provided: 3 prime UTR variants (teal), missense variants (yellow), splice region variants (red), synonymous variants (green), intron variants (dark blue).

© 2021 Jha RM et al. *JAMA Network Open*.

eReferences

1. Jha RM, Puccio AM, Okonkwo DO, et al. (2017) ABCC8 Single Nucleotide Polymorphisms are Associated with Cerebral Edema in Severe TBI. Neurocrit Care 26:213–224. doi: 10.1007/s12028-016-0309-z
2. Jha RM, Koleck TA, Puccio AM, et al. (2018) Regionally clustered ABCC8 polymorphisms in a prospective cohort predict cerebral oedema and outcome in severe traumatic brain injury. J Neurol Neurosurg Psychiatry 89:1152–1162. doi: 10.1136/jnnp-2017-317741
3. Jha RM, Desai SM, Zusman BE, et al. (2019) Downstream TRPM4 Polymorphisms Are Associated with Intracranial Hypertension and Statistically Interact with ABCC8 Polymorphisms in a Prospective Cohort of Severe Traumatic Brain Injury. J Neurotrauma 36:1804–1817. doi: 10.1089/neu.2018.6124
4. Jha RM, Elmer J, Zusman BE, et al. (2018) Intracranial pressure trajectories: A novel approach to informing severe traumatic brain injury phenotypes. Crit Care Med 46:1792–1802. doi: 10.1097/CCM.0000000000003361
5. Kothari RU, Brott T, Broderick JP, et al. (1996) The ABCs of measuring intracerebral hemorrhage volumes. Stroke 27:1304–1305. doi: 10.1161/01.STR.27.8.1304
6. Adatia K, Newcombe VFJ, Menon DK (2020) Contusion progression following traumatic brain injury: A review of clinical and radiological predictors, and influence on outcome. Neurocrit Care. doi: 10.1007/s12028-020-00994-4
7. GTEx Consortium, Laboratory, Data Analysis &Coordinating Center (LDACC)—Analysis Working Group, Statistical Methods groups—Analysis Working Group, et al. (2017) Genetic effects on gene expression across human tissues. Nature 550:204–213. doi: 10.1038/nature24277
8. Ward LD, Kellis M (2016) HaploReg v4: systematic mining of putative causal variants, cell types, regulators and target genes for human complex traits and disease. Nucleic Acids Res 44:D877-81. doi: 10.1093/nar/gkv1340
9. Ward LD, Kellis M (2012) HaploReg: a resource for exploring chromatin states, conservation, and regulatory motif alterations within sets of genetically linked variants. Nucleic Acids Res 40:D930-4. doi: 10.1093/nar/gkr917
10. Boyle AP, Hong EL, Hariharan M, et al. (2012) Annotation of functional variation in personal genomes using RegulomeDB. Genome Res 22:1790–1797. doi: 10.1101/gr.137323.112
11. Yates AD, Achuthan P, Akanni W, et al. (2020) Ensembl 2020. Nucleic Acids Res 48:D682–D688. doi: 10.1093/nar/gkz966
12. Autzen HE, Myasnikov AG, Campbell MG, et al. (2018) Structure of the human TRPM4 ion channel in a lipid nanodisc. Science 359:228–232. doi: 10.1126/science.aar4510
13. Berman HM, Westbrook J, Feng Z, et al. (2000) The protein data bank. Nucleic Acids Res 28:235–242. doi:

© 2021 Jha RM et al. *JAMA Network Open*.

10.1093/nar/28.1.235
14. Li N, Wu J-X, Ding D, et al. (2017) Structure of a Pancreatic ATP-Sensitive Potassium Channel. Cell 168:101–110.e10. doi: 10.1016/j.cell.2016.12.028
15. Martin GM, Yoshioka C, Rex EA, et al. (2017) Cryo-EM structure of the ATP-sensitive potassium channel illuminates mechanisms of assembly and gating. Elife. doi: 10.7554/eLife.24149
16. Pettersen EF, Goddard TD, Huang CC, et al. (2004) UCSF Chimera—a visualization system for exploratory research and analysis. J Comput Chem 25:1605–1612. doi: 10.1002/jcc.20084

© 2021 Jha RM et al. *JAMA Network Open*.
